# Supplementary material for: Design and structural validation of peptide–drug conjugate ligands of the kappa-opioid receptor
Source: Nat Commun. 2023 Dec 6;14:8064. doi: 10.1038/s41467-023-43718-w (PMC10698194; doi:10.1038/s41467-023-43718-w)
Supplement: Supplementary file 1 — Supplementary Information [file 41467_2023_43718_MOESM1_ESM.pdf]

Supplementary Information

De novo design and structural validation of peptide–drug conjugate ligands of the kappa-opioid receptor

Edin Muratspahić[#], Kristine Deibler[#], Jianming Han[#], Nataša Tomašević, Kirtikumar B. Jadhav, Aina-Leonor Olivé-Marti, Nadine Hochrainer, Roland Hellinger, Johannes Koehbach, Jonathan F. Fay, Mohammad Homaidur Rahman, Lamees Hegazy, Timothy Craven, Balazs R. Varga, Gaurav Bhardwaj, Kevin Appourchaux, Susruta Majumdar, Markus Muttenthaler, Parisa Hosseinzadeh, David J. Craik, Mariana Spetea, Tao Che[*], David Baker[*], Christian W. Gruber[*]

[#]These authors contributed equally.

*Correspondence to: Tao Che (email: taoche@wustl.edu), David Baker (email: dabaker@uw.edu), Christian W. Gruber (email: christian.w.gruber@meduniwien.ac.at)

This PDF file includes:

Supplementary Figs. 1 to 30

Supplementary Tables 1 to 15

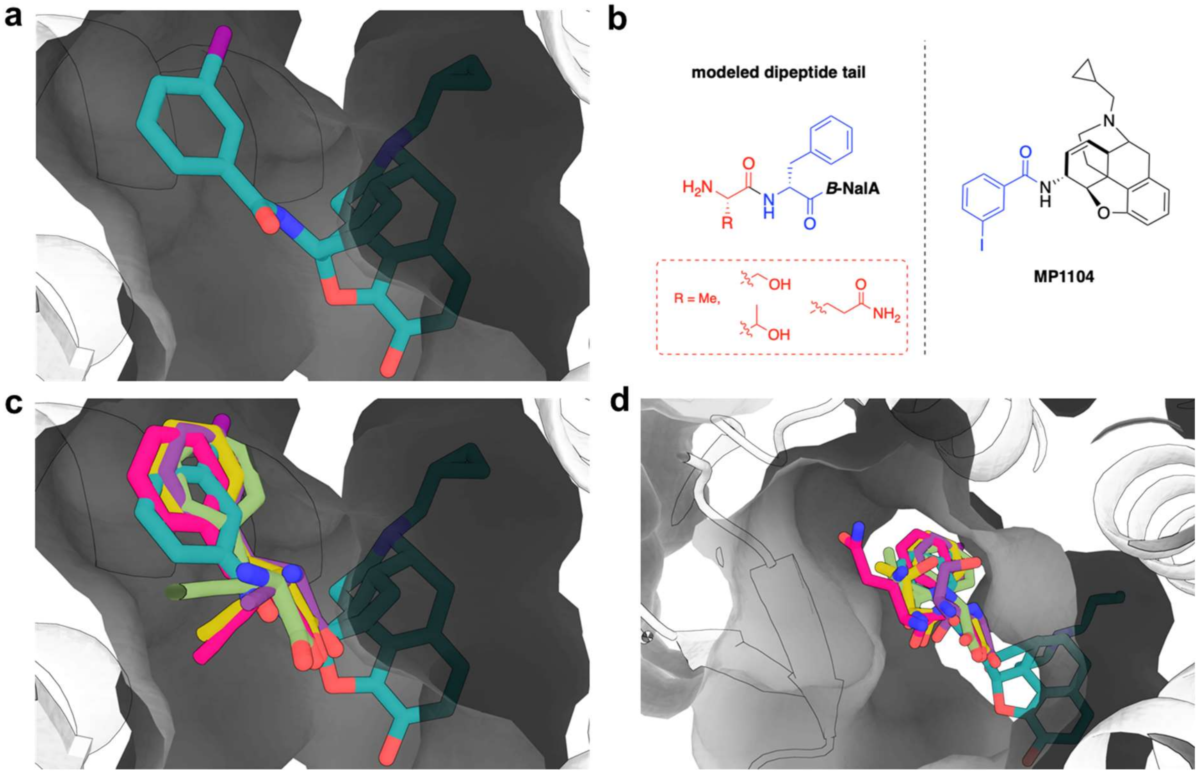

Supplementary Fig. 1 | Mimicking of the iodobenzamide group of MP1104 with a phenylalanine. **a,** MP1104 in the binding with view of the iodobenzamide. **b,** Schematic of MP1104 and the structural moieties being mimicked by D-Phe and an additional amino acid (color coordinated). **c,** D-Phe mimics the iodobenzamide group of MP1104 allowing for maintained hydrophobic pocket interaction. **d,** Further extension of the lariat tail was accomplished through sampling torsion angles and side chains of one additional amino acid.

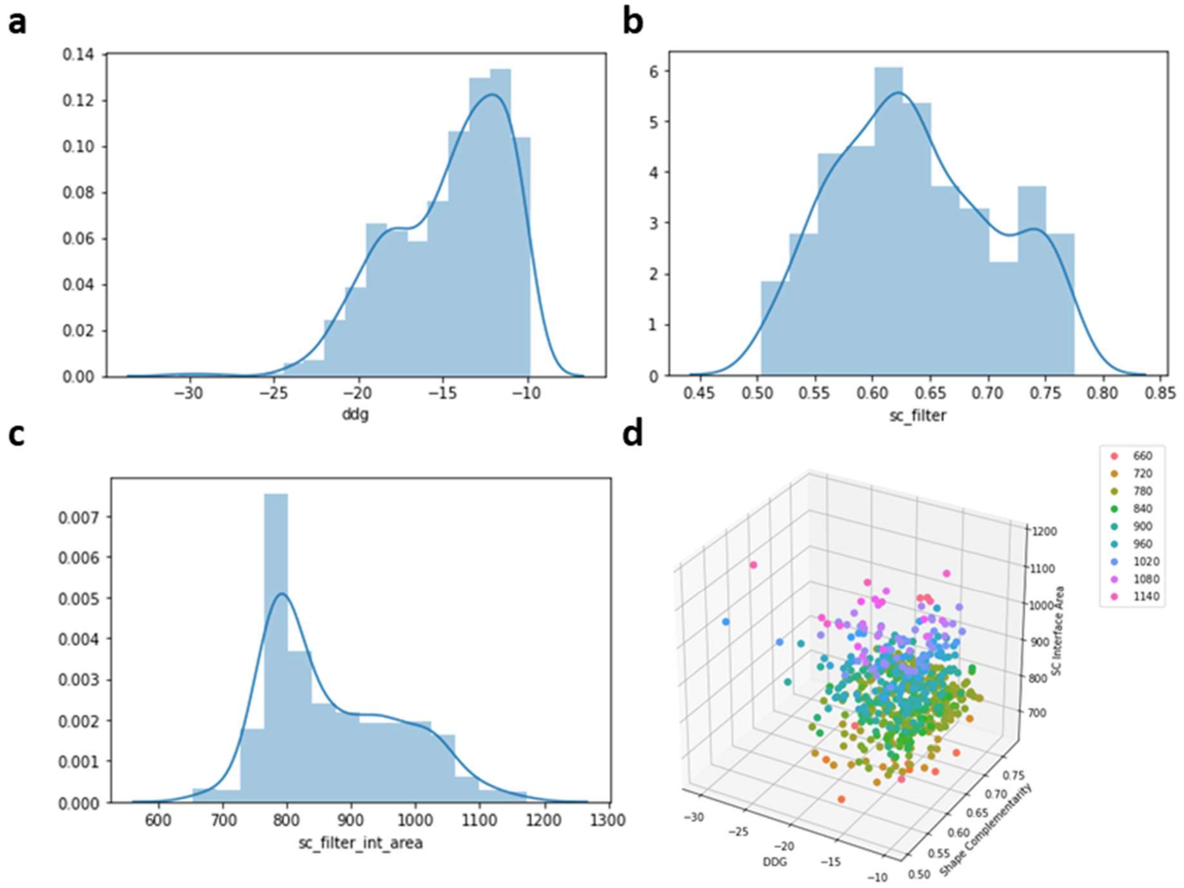

Supplementary Fig. 2 | Histograms of select metrics of the output designs for 6-mers. a, ΔΔG **b,** Shape complementarity filter (sc_filter) **c,** Shape complementarity Interface Area. **d,** 3D plot of ΔΔG, shape complementarity and Interface area; ΔΔG < -19.4, shape_complementarity > 0.74, shape_complementarity_interface_area > 1028.

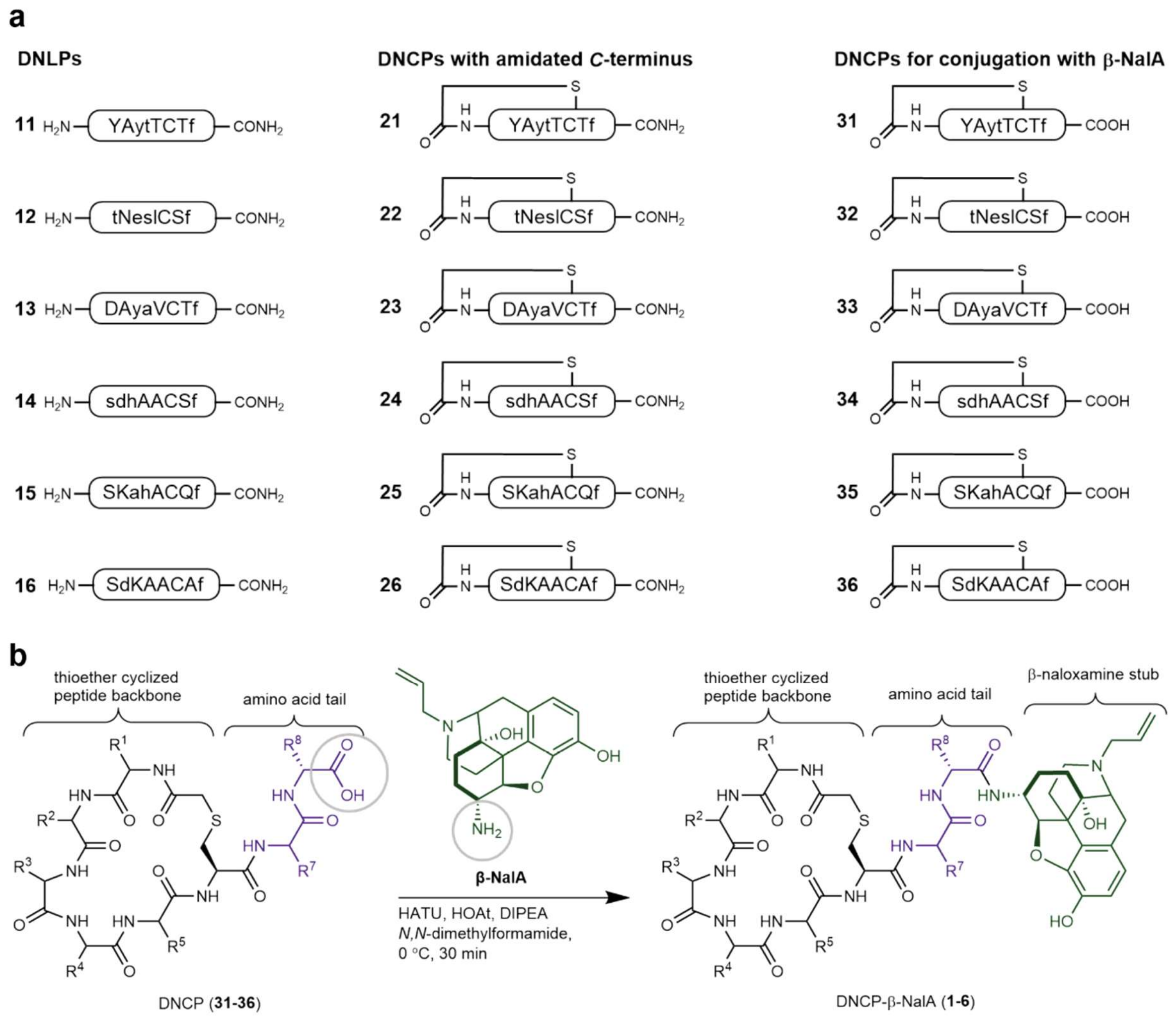

Supplementary Fig. 3 | Structures of de novo linear peptides (DNLPs) and de novo cyclic peptides (DNCPs) and conjugation reactions of DNCPs with β-naloxamine (β-NalA). a, Structures of de novo designed and synthesized peptide libraries of DNLPs and DNCPs, where capital letter = L-amino acid, small letters = D-amino acids, peptide N-terminal groups are explicitly labelled as amine (NH_2) and C-terminal groups as carbonyl groups (COOH/$CONH_2$). **b,** Conjugation reactions of DNCP (**31-36**) with β-naloxamine (β-NalA), where R# denotes amino acid side chain described in (**a**).

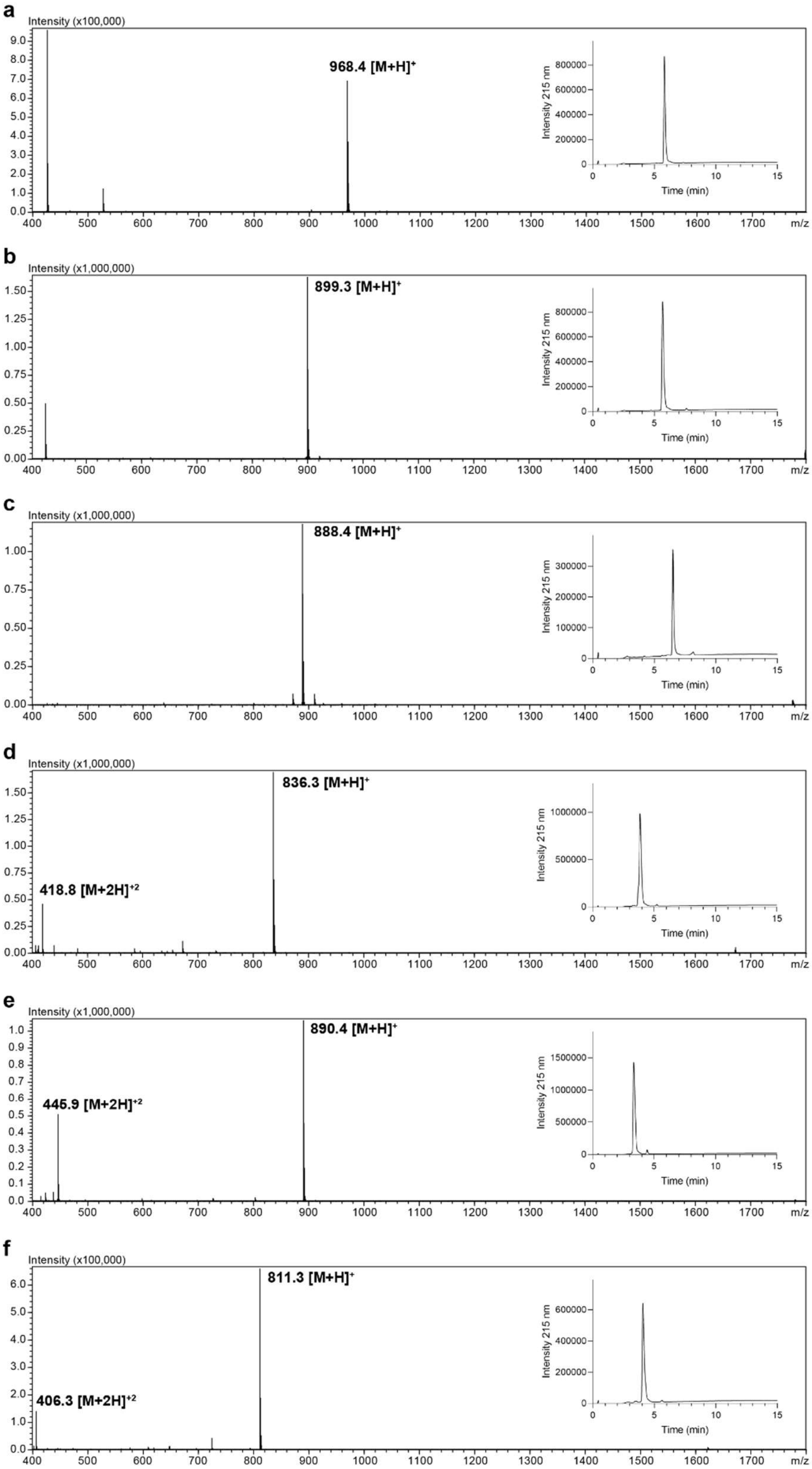

Supplementary Fig. 4 | Quality control of de novo linear peptides (DNLP) (11-16). a-f, Purity of DNLP (**11**) (**a**), DNLP (**12**) (**b**), DNLP (**13**) (**c**), DNLP (**14**) (**d**), DNLP (**15**) (**e**) and DNLP (**16**) (**f**) (calculated by automatic peak integration from 5-15 min) was determined by analytical RP-UPLC (insets) using a Phenomenex Luna Omega C_{18} column (1.6 µm, 100 Å, 50 x 2.1 mm) with a linear gradient of 1-61% of solvent B (90% acetonitrile, 10% H_2O, 0.05% trifluoroacetic acid) in 15 min and a flowrate of 0.6 mL min^{-1}. Monoisotopic masses ($[M+H]^+$) and ($[M+2H]^{2+}$) of DNLPs were identified by ESI-MS on a LCMS-2020. All DNLPs had a purity >95% except DNLP (**13**) which had purity of 94%. Peptides were synthesized with an amidated C-terminus.

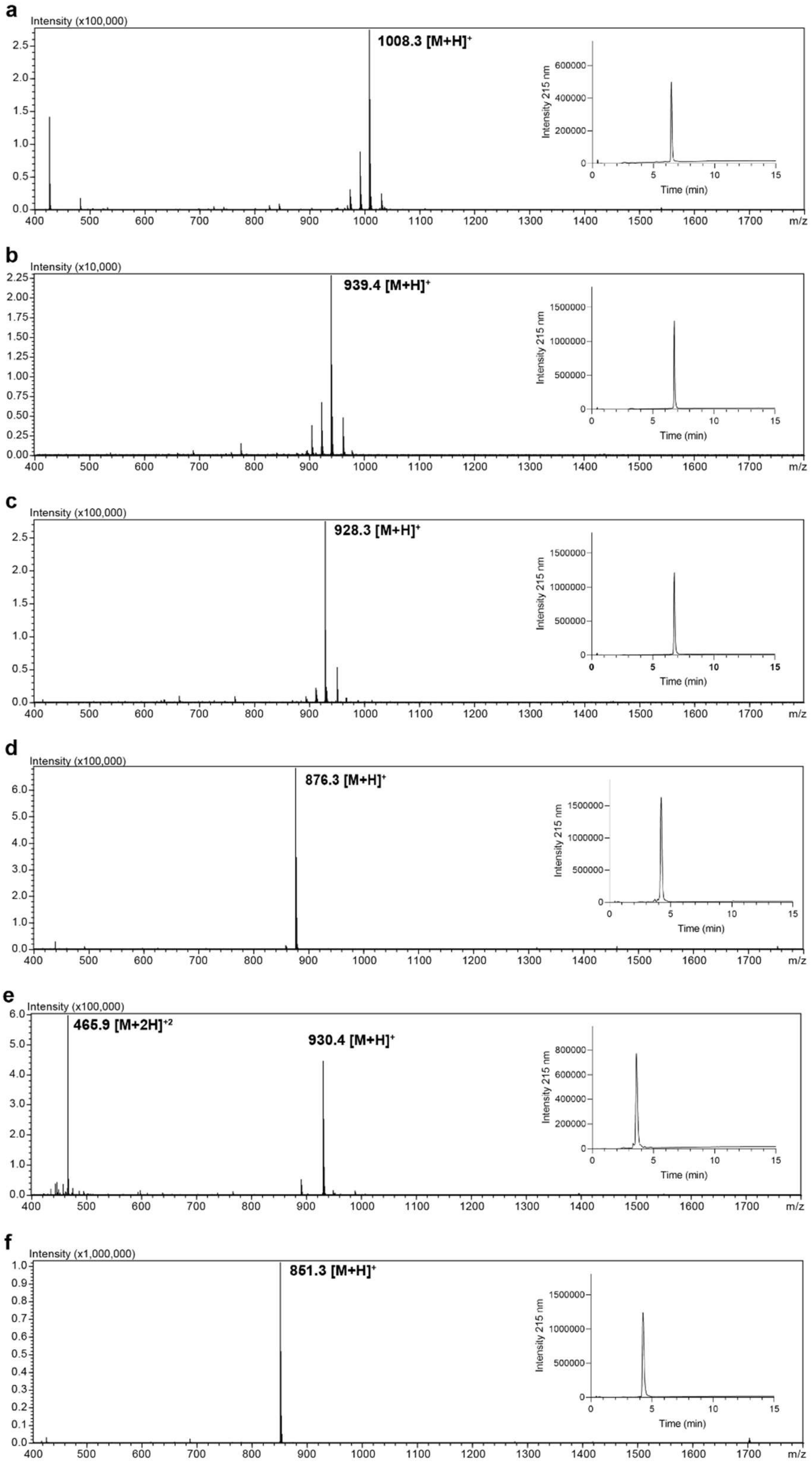

Supplementary Fig. 5 | Quality control of *de novo* cyclic (DNCP) peptides (21-26). a-f, Purity of DNCP (**21**) (**a**), DNCP (**22**) (**b**), DNCP (**23**) (**c**), DNCP (**24**) (**d**), DNCP (**25**) (**e**) and DNCP (**26**) (**f**) (calculated by automatic peak integration from 5-15 min) was determined by analytical RP-UPLC (insets) using a Phenomenex Luna Omega C_{18} column (1.6 µm, 100 Å, 50 x 2.1 mm) with a linear gradient of 1-61% of solvent B (90% acetonitrile, 10% H_2O, 0.05% trifluoroacetic acid) in 15 min and a flowrate of 0.6 mL min^{-1}. Monoisotopic masses ($[M+H]^+$) and ($[M+2H]^{2+}$) of DNCPs were identified by ESI-MS on a LCMS-2020 system. All DNCPs had a purity >95%. Peptides were synthesized with an amidated C-terminus.

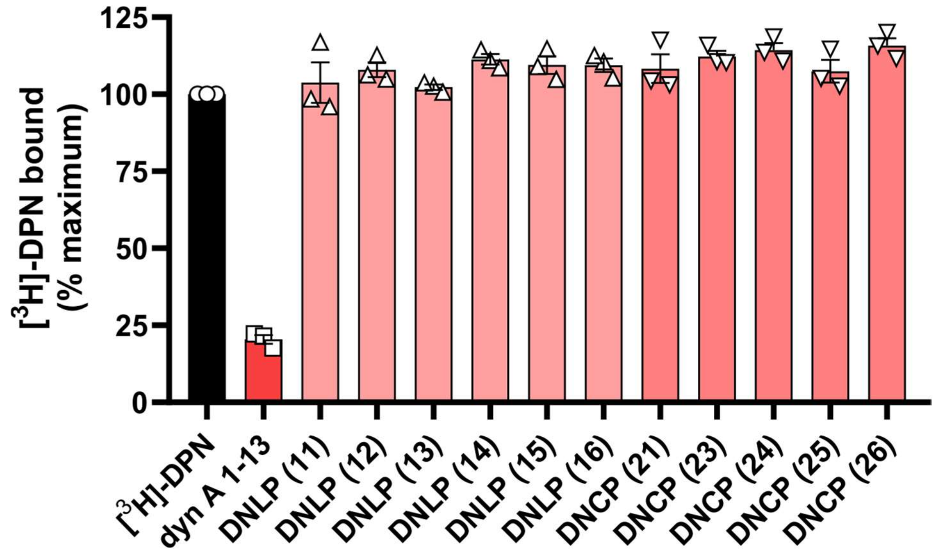

Supplementary Fig. 6 | Determination of specific binding of DNLPs (11-16) and DNCPs (21-26) at the mouse KOR. The ability of peptides (10 μM) to displace [^{3}H]diprenorphine ([^{3}H]DPN, 1 nM) was measured in the radioligand binding assay using membranes from HEK203 cells stably expressing the mouse KOR. Dynorphin (dyn) $A_{1\text{-}13}$ was used as positive control (10 nM). Specific binding was calculated by subtracting total (determined in the absence of test peptide) from non-specific binding (determined in the presence of naloxone 10 μM). We were not able to evaluate the affinity of DNCP (22) in the radioligand binding assay since this peptide did not dissolve in water or in organic solvents such as DMSO. Data are presented as the mean ± s.e.m. from three independent experiments.

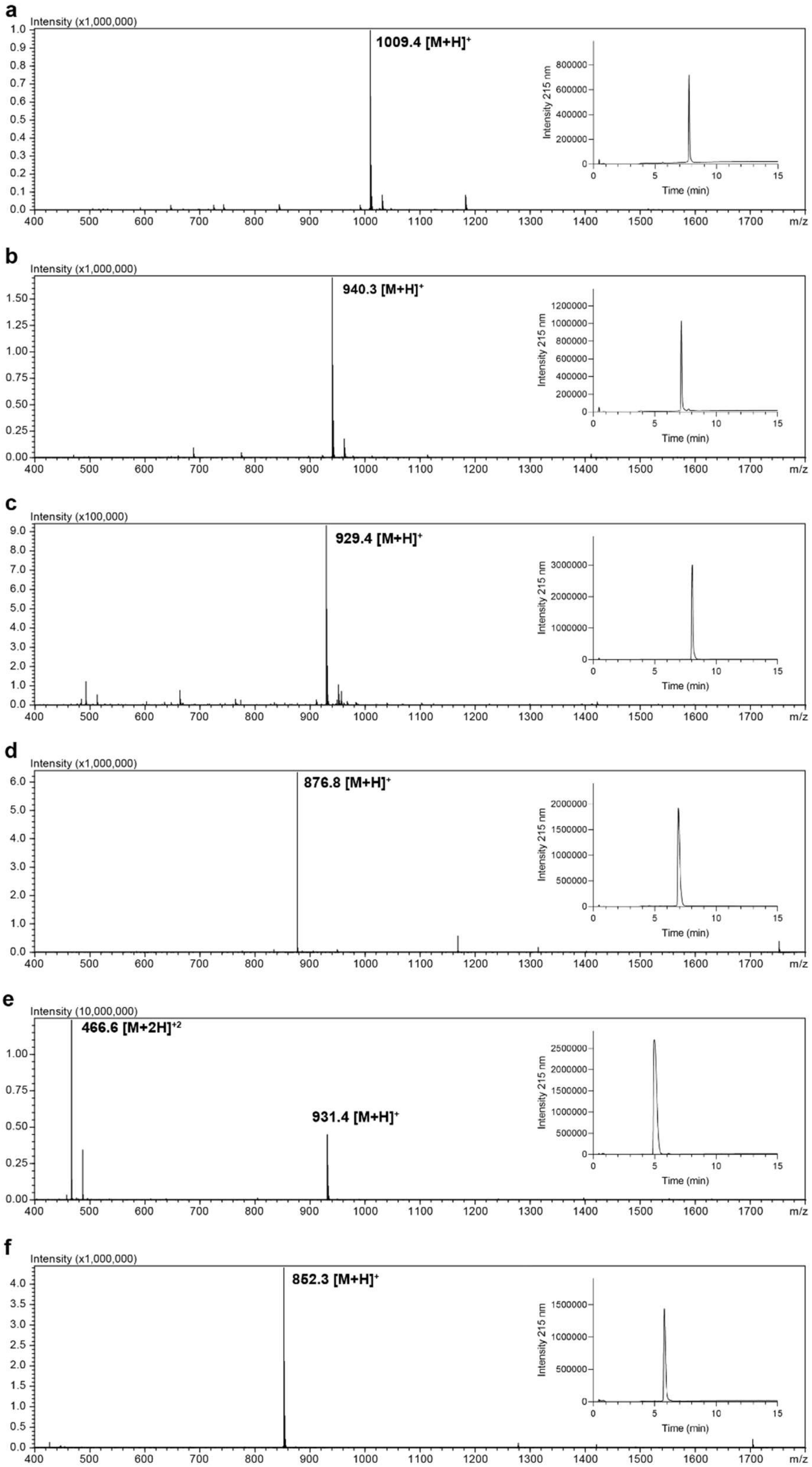

Supplementary Fig. 7 | Quality control of de novo cyclic peptides (DNCP) (31-36) used for conjugation. a-f, Purity of DNCP (**31**) (**a**), DNCP (**32**) (**b**), DNCP (**33**) (**c**), DNCP (**34**) (**d**), DNCP (**35**) (**e**) and DNCP (**36**) (**f**) (calculated by automatic peak integration from 5-15 min) was determined by analytical RP-UPLC (insets) using a Phenomenex Luna Omega C_{18} column (1.6 µm, 100 Å, 50 x 2.1 mm) with a linear gradient of 1-61% of (90% acetonitrile, 10% H_2O, 0.05% trifluoroacetic acid) in 15 min and a flowrate of 0.6 mL min^{-1}. Monoisotopic masses ($[M+H]^+$) and ($[M+2H]^{2+}$) of DNCPs were identified by ESI-MS on a LCMS-2020. All DNCPs had a purity >95%. Peptides were synthesized with the COOH at the C-terminus.

a

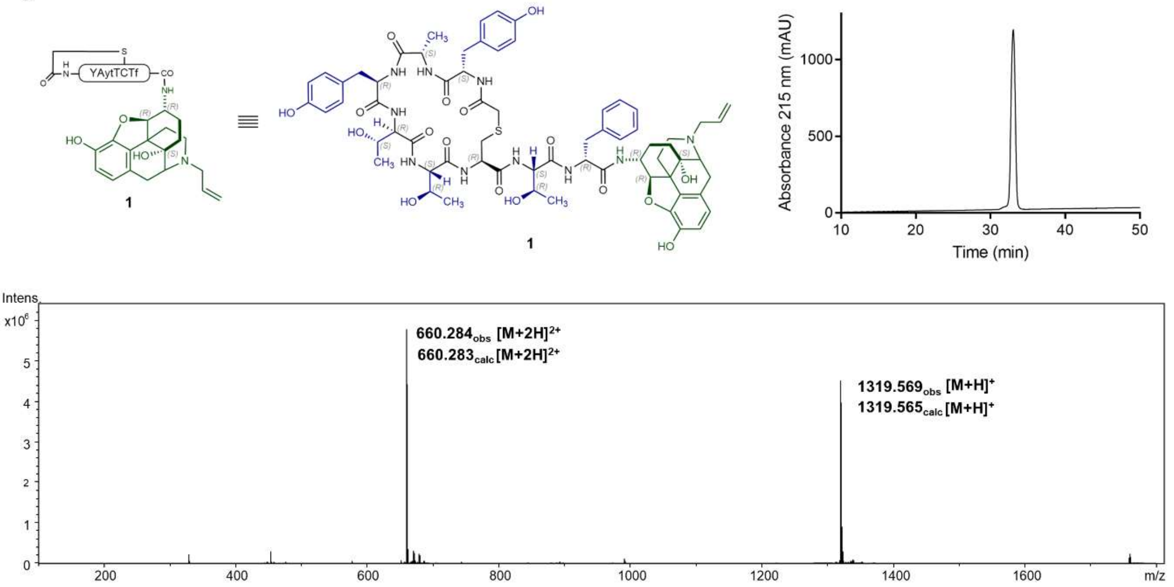
YAytTCTf
1
Absorbance 215 nm (mAU)
Time (min)
Intens.
x10^6
660.284obs [M+2H]2+
660.283calc [M+2H]2+
1319.569obs [M+H]+
1319.565calc [M+H]+
m/z

b

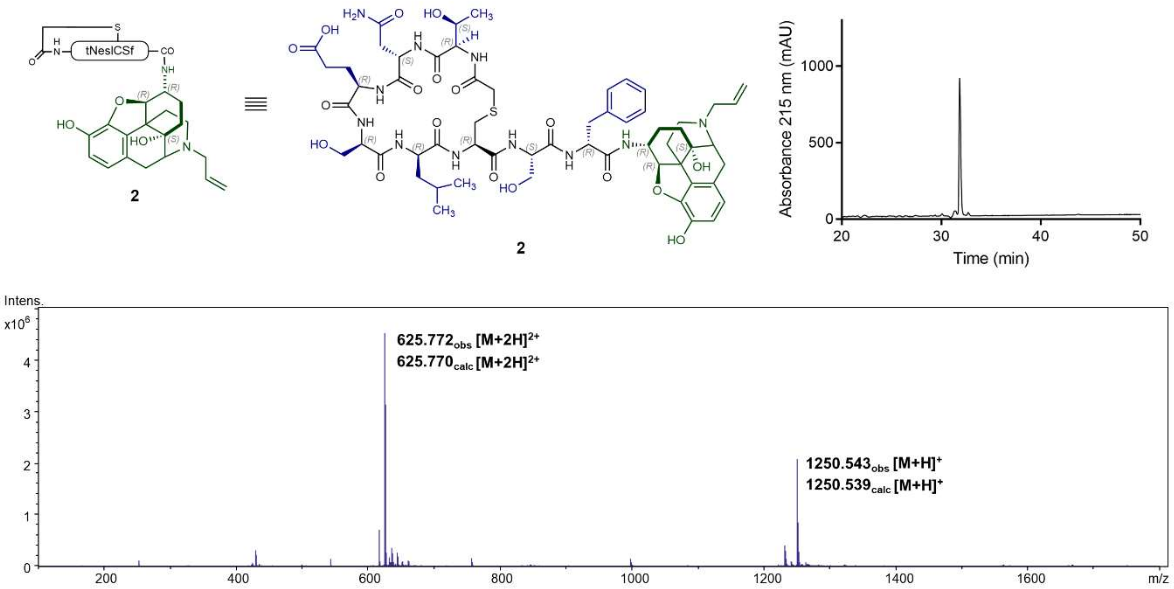
tNesICSf
2
Absorbance 215 nm (mAU)
Time (min)
Intens.
x10^6
625.772obs [M+2H]2+
625.770calc [M+2H]2+
1250.543obs [M+H]+
1250.539calc [M+H]+
m/z

c

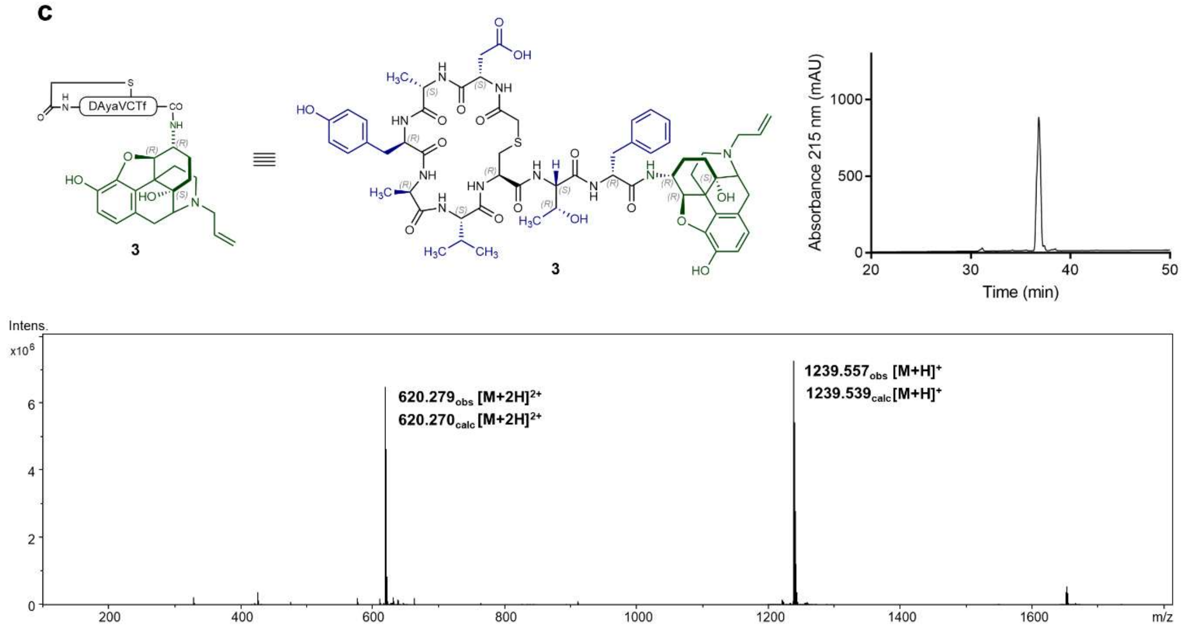

d

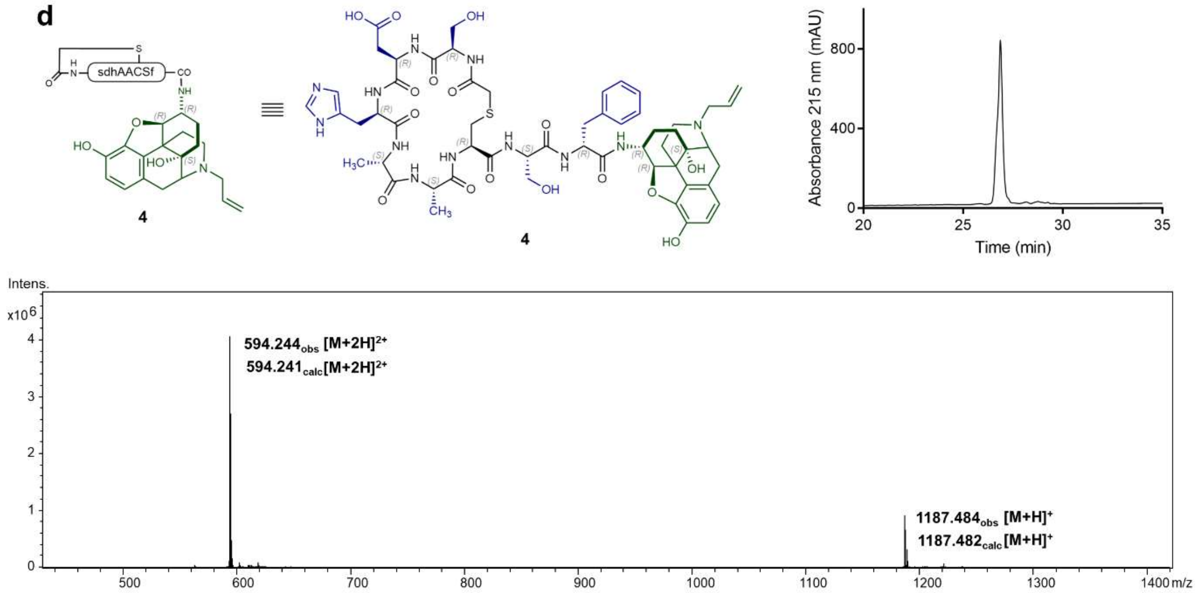

Supplementary Fig. 8 | Analytical characterization of *de novo* cyclic peptides (DNCP)-β-NalA conjugates (1-4). a-d, 1D structures (left), RP-HPLC (right) and MALDI-MS (bottom) of DNCP-β-NalA(**1**) (**a**), DNCP-β-NalA(**2**) (**b**), DNCP-β-NalA(**3**) (**c**) and DNCP-β-NalA(**4**) (**d**) are shown. RP-HPLC analyses were performed on a RP-HPLC using a Phenomenex Jupiter C_{18} column (5 μM, 300 Å, 50 × 2 mm) and a linear gradient of 5-65% solvent B (90% acetonitrile, 10% H_2O, 0.1% trifluoroacetic acid) in 60 min at a flow rate of 1 mL min^{-1}. Monoisotopic masses ($[M+H]^+$) and ($[M+2H]^{2+}$ were identified by ESI-MS on a LC-QqTOF compact. Observed (obs) and calculated (calc) monoisotopic masses are shown.

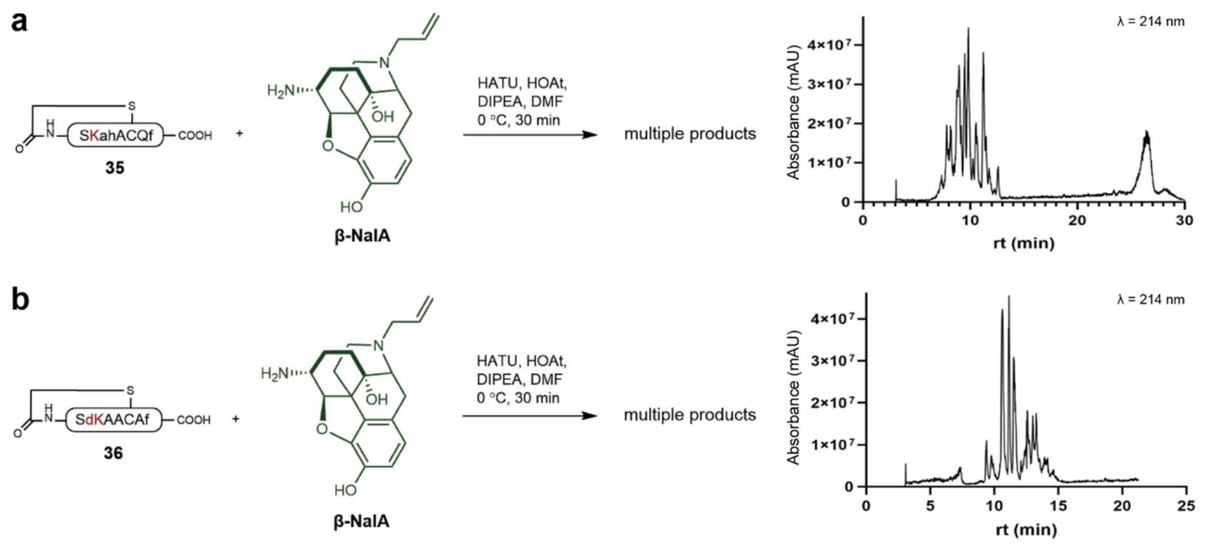

Supplementary Fig. 9 | Unsuccessful synthesis of two of the *de novo* cyclic peptides (DNCP)-β-NalA conjugates (5-6). a-b, Conjugation reaction of DNCP (**35 & 36**) with β-NalA to produce DNCP-β-NalA(**5**) (**a**) and DNCP-β-NalA(**6**) (**b**) was non-selective because of Lys and Asp present in the sequence, which led to the formation of an inseparable mixture of multiple products.

```
                                     NT
hKOR   MDSPIQIFRGEPGPTCAPSACLPPNSSAWFPGWAEPDSNGSAGSEDAQLEPAHISPAIPV   60
mKOR   MESPIQIFRGDPGPTCSPSACLLPNSSSWFPNWAESDSNGSVGSEDQQLESAHISPAIPV   60

                  TM1               ICL1              TM2
hKOR   IITAVYSVVFVVGLVGNSLVMFVIIRYTKMKTATNIYIFNLALADALVTTTMPFQSTVYL   120
mKOR   IITAVYSVVFVVGLVGNSLVMFVIIRYTKMKTATNIYIFNLALADALVTTTMPFQSAVYL   120

           ECL1                TM3                    ICL2
hKOR   MNSWPFGDVLCKIVISIDYYNMFTSIFTLTMMSVDRYIAVCHPVKALDFRTPLKAKIINI   180
mKOR   MNSWPFGDVLCKIVISIDYYNMFTSIFTLTMMSVDRYIAVCHPVKALDFRTPLKAKIINI   180

             TM4                  ECL2                    TM5
hKOR   CIWLLSSSVGISAIVLGGTKVREDVDVIECSLQFPDDDYSWWDLFMKICVFIFAFVIPVL   240
mKOR   CIWLLASSVGISAIVLGGTKVREDVDVIECSLQFPDDEYSWWDLFMKICVFVFAFVIPVL   240

                            ICL3                  TM6
hKOR   IIIVCYTLMILRLKSVRLLSGSREKDRNLRRITRLVLVVVAVFVVCWTPIHIFILVEALG   300
mKOR   IIIVCYTLMILRLKSVRLLSGSREKDRNLRRITKLVLVVVAVFIICWTPIHIFILVEALG   300

          ECL3             TM7                    CT
hKOR   STSHSTAALSSYYFCIALGYTNSSLNPILYAFLDENFKRCFRDFCFPLKMRMERQSTSRV   360
mKOR   STSHSTAALSSYYFCIALGYTNSSLNPVLYAFLDENFKRCFRDFCFPIKMRMERQSTNRV   360

hKOR   RNTVQDPAYLRDIDGMNKPV   380
mKOR   RNTVQDPASMRDVGGMNKPV   380
```

Supplementary Fig. 10 | Comparison of the amino acid sequences of human and mouse KOR. Transmembrane domains are colored in grey. Transmembrane domains (TM1–7), extra- (ECL1-3) and intracellular loops (ICL1–3), N- and C-terminal tail (CT) are labeled above the domain, and amino acid residues are indicated on the right. Sequence differences are highlighted in red.

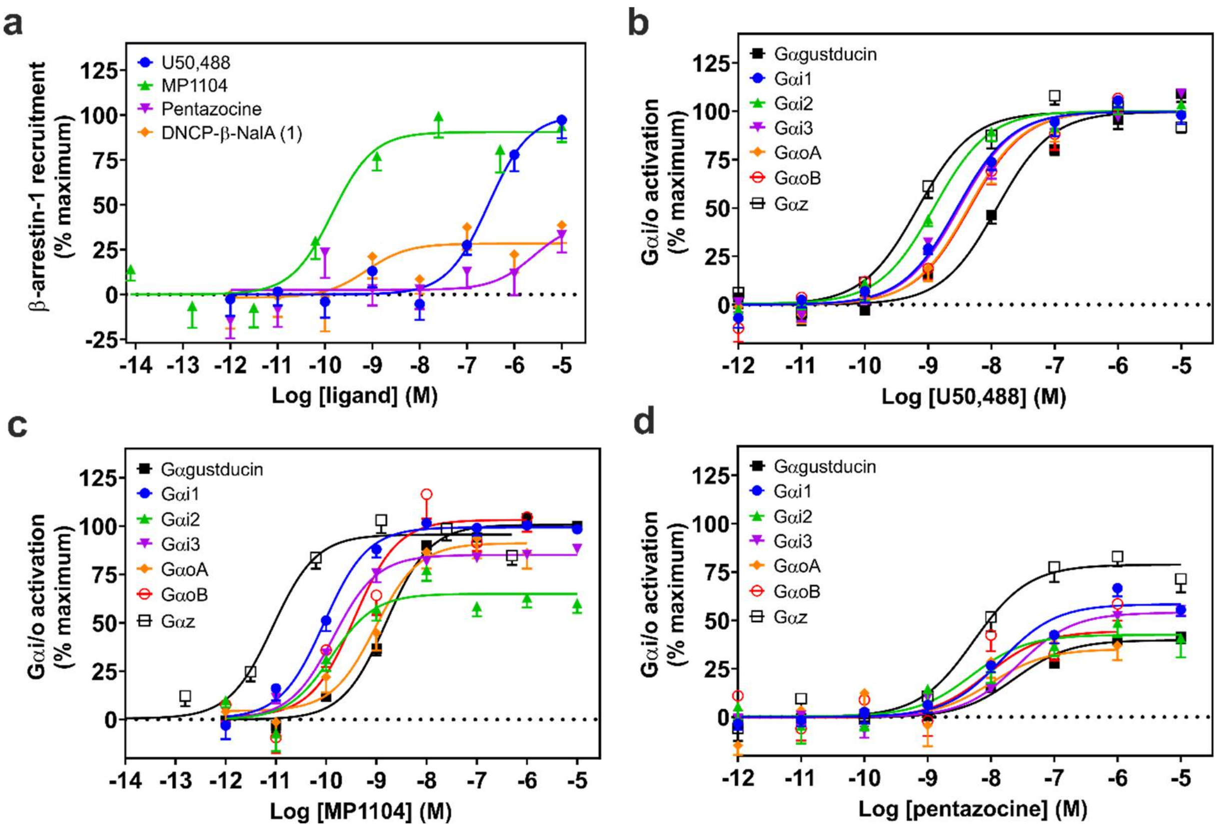

Supplementary Fig. 11 | β-arrestin-1 recruitment and TRUPATH assay data. a, β-arrestin-1 screening of lead peptide DNCP-β-NalA(**1**) and other KOR ligands as controls, i.e., U50,488, MP1104 and pentazocine**. b-d,** G*α*-subtype screening of U50,488 (**b**), MP1104 (**c**) and pentazocine (**d**) at the human KOR in the TRUPATH assay. Data are shown as the mean ± s.e.m. from four independent experiments and are normalized to U50,488.

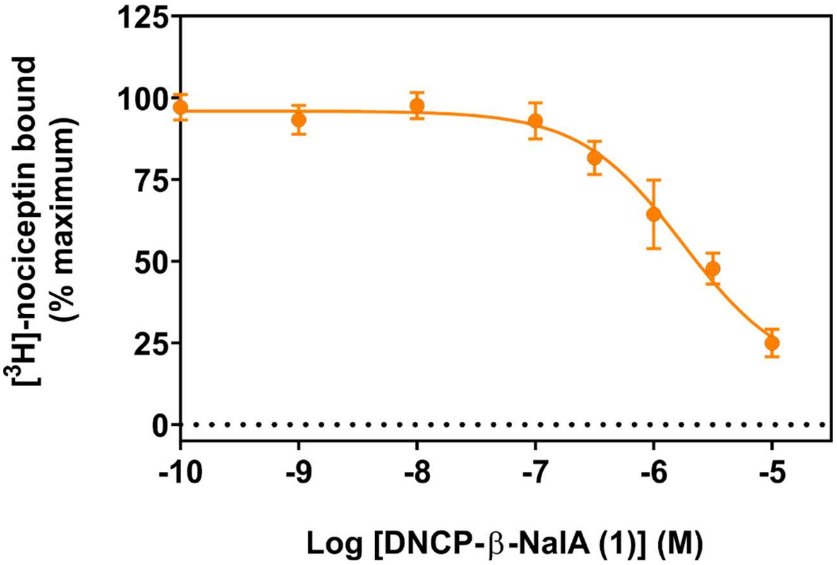

Supplementary Fig. 12 | Binding of DNCP-β-NalA (1) at human NOP receptor. Binding of DNCP-β-NalA(**1**) at human nociceptin (NOP) receptor was determined in a radioligand binding assay using CHO cell membrane preparations stably expressing human NOP receptor and in the presence of 0.1 nM of [^{3}H]nociceptin. Data are shown as the mean ± s.e.m. from six independent experiments.

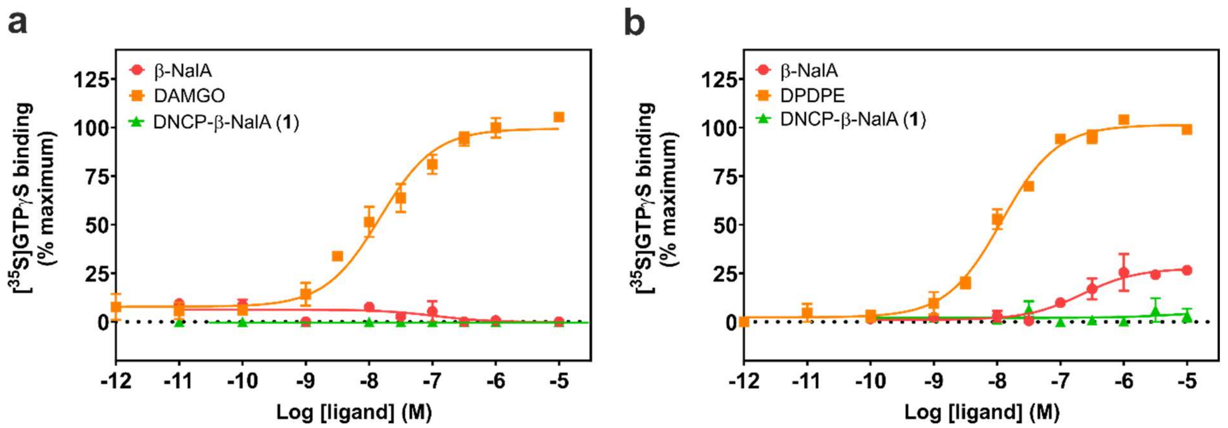

Supplementary Fig. 13 | [^{35}S]GTPγS binding assays at the human MOR and DOR. a, Concentration-dependent stimulation of [^{35}S]GTPγS binding by DNCP-β-NalA(**1**), β-NalA and DAMGO using membranes from CHO-hMOR cells. **b,** Concentration-dependent stimulation of [^{35}S]GTPγS binding by DNCP-β-NalA(**1**), β-NalA and DPDPE using membranes from CHO-hDOR cells. Percentage stimulation is presented relative to the maximum simulation of reference full agonist DAMGO (MOR) and DPDPE (DOR). Data are presented as the mean ± s.e.m. from three independent experiments.

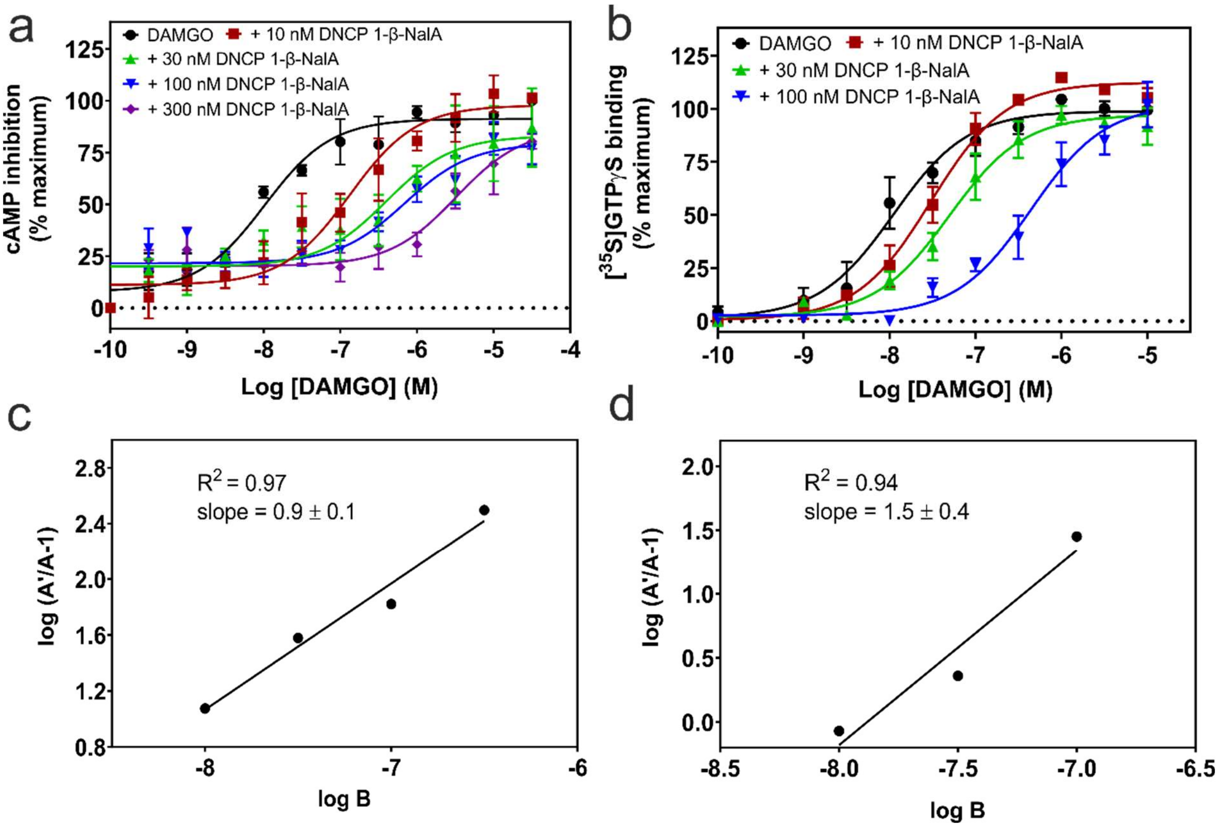

Supplementary Fig. 14 | Competitive antagonism of DNCP-β-NalA(1) at the MOR. a, Concentration-response curves of DAMGO in the absence and presence of 10, 30, 100 and 300 nM of DNCP-β-NalA(**1**) in cAMP assay was measured in HEK293 cells stably expressing mouse MOR. **b,** Concentration-response curves of DAMGO in the absence (n=5) and presence of 10 nM (n=3), 30 nM (n=3) and 100 nM (n=3) of DN*CP*-β-NalA(**1**) in [35S]GTPγS binding assay was measured in membranes from CHO cells stably expressing human MOR. Data were normalized to the percentage of maximum activation of DAMGO, and represent the mean ± s.e.m . Schild regression analysis of DNCP-β-NalA(**1**) in **c,** cAMP and **d,** [35S]GTPγS binding assay. A represents EC_{50} of DAMGO in presence of DNCP-β-NalA(**1**), A′ is EC_{50} of DAMGO, and B represents logarithm of concentration of DNCP-β-NalA(**1**).

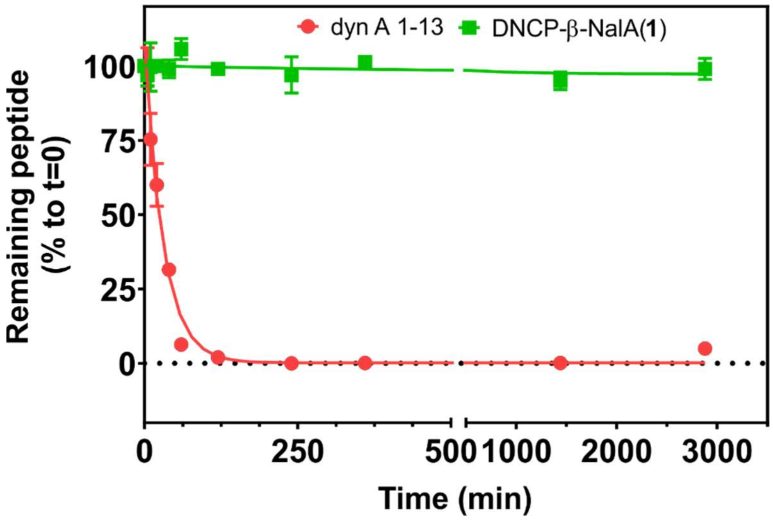

Supplementary Fig. 15 | Serum stability of DNCP-β-NalA(1). Stability of DNCP-β-NalA(**1**) and dyn $A_{1\text{-}13}$ was measured in human serum at indicated time points using the HPLC system (mean ± s.e.m., n=3). A Phenomenex Kinetex C_{18} (150 × 3 mm, 2.6 μm, 110 Å) column was used with mobile phases 0.1% trifluoroacetic acid in water and acetonitrile/water trifluoroacetic acid 90/10/0.1% (v/v/v) with a linear separation gradient over 30 min.

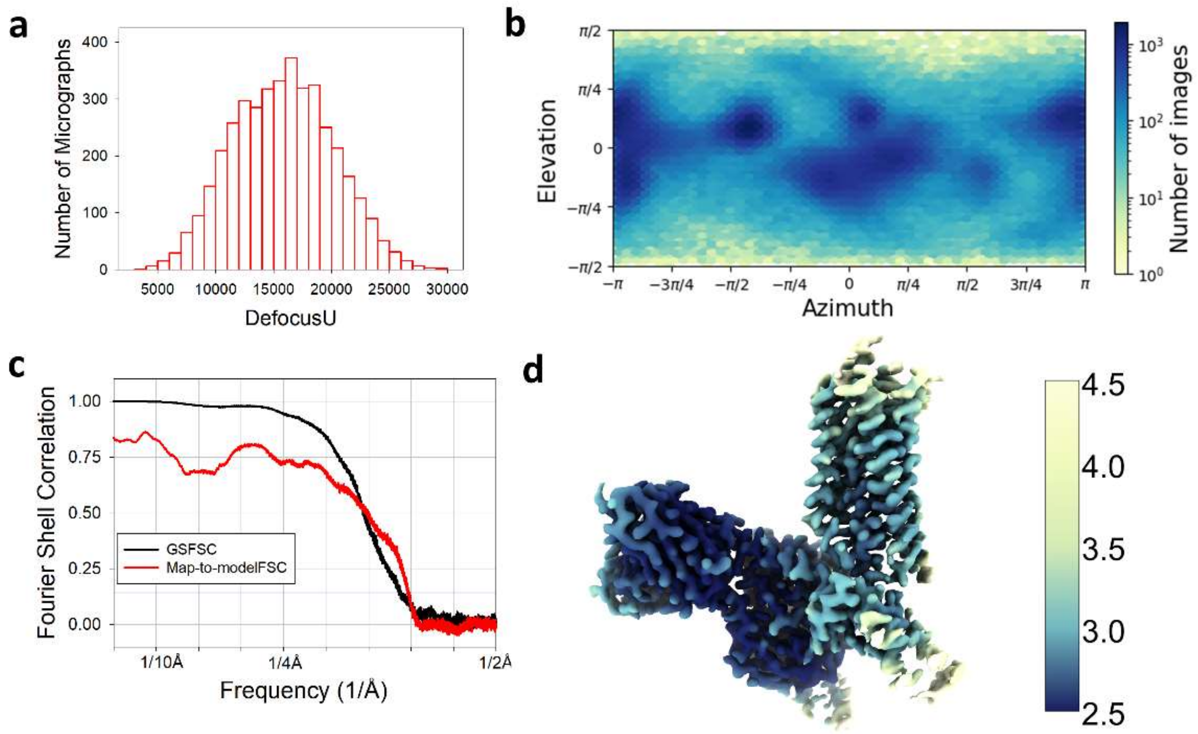

Supplementary Fig. 16 | Cryo-EM data processing of KOR-DNCP-β-NalA(1)-G_{i1} complex. a, Histograms of defocus values for micrographs used in the single-particle analysis. **b,** Orientational distribution heat map. **c,** 2D plots of the gold-standard Fourier shell correlation (GSFSC) between half maps (black) and FSC between model and the B-factor sharpened map for respective refined model (red) as calculated by Phenix_mtriage. **d,** Local resolution heat-map calculated using the local windowed FSC method.

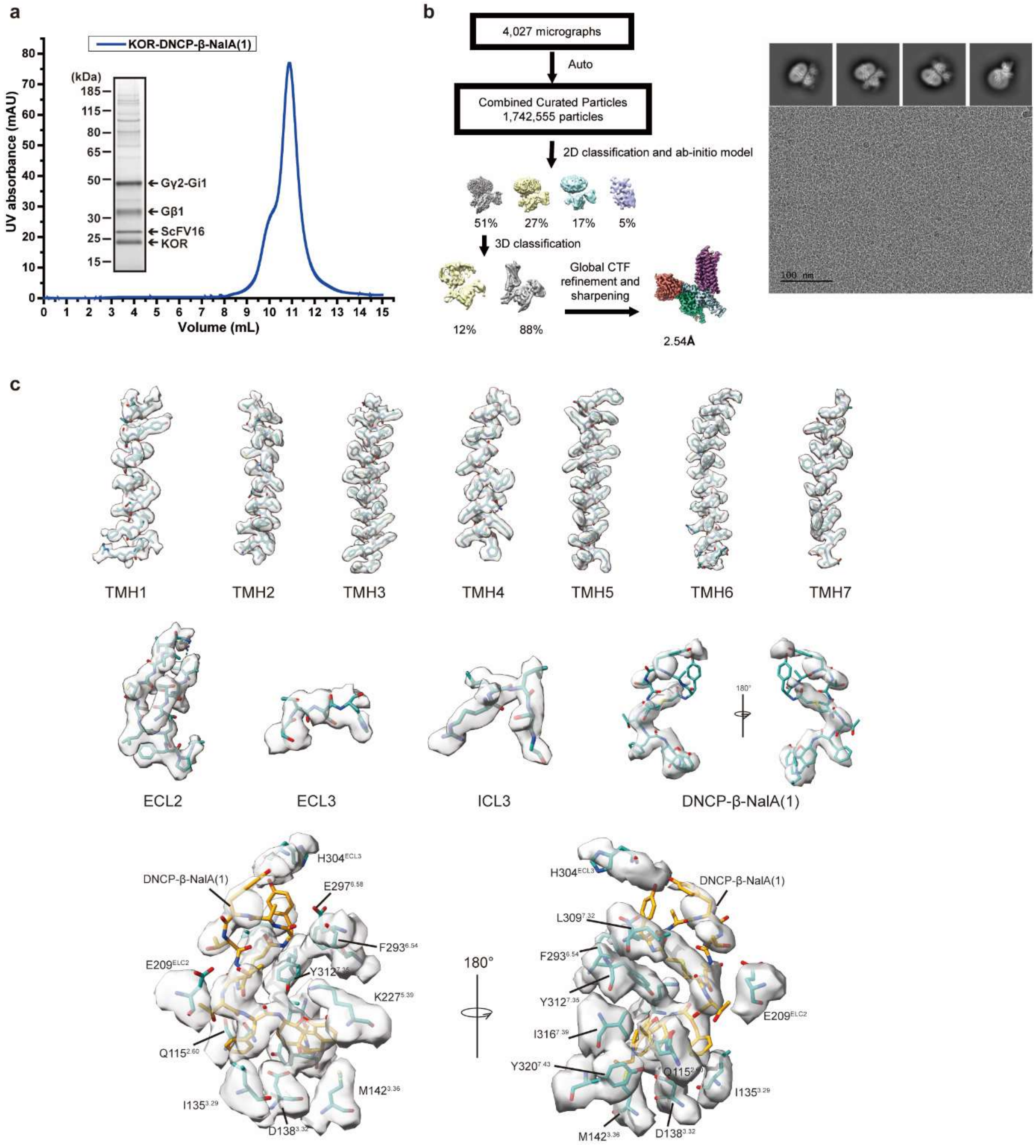

Supplementary Fig. 17 | Cryo-EM data reconstruction of KOR-DNCP-β-NalA(1)-G_{i1} complex. a, Size-exclusion chromatogram and SDS-PAGE analysis of the purified KOR-DNCP-β-NalA(**1**)-G_{i1} complex. **b,** Cryo-EM data processing flowcharts of the KOR-DNCP-β-NalA(**1**)-G_{i1} complex. **c,** Cryo-EM density of all seven transmembrane helices (TMH1-7), extracellular loop 2 and 3 (ECL2 and 3), intracellular loop 3 (ICL3), ligand DNCP-β-NalA(**1**) and ligand-binding pocket residues.

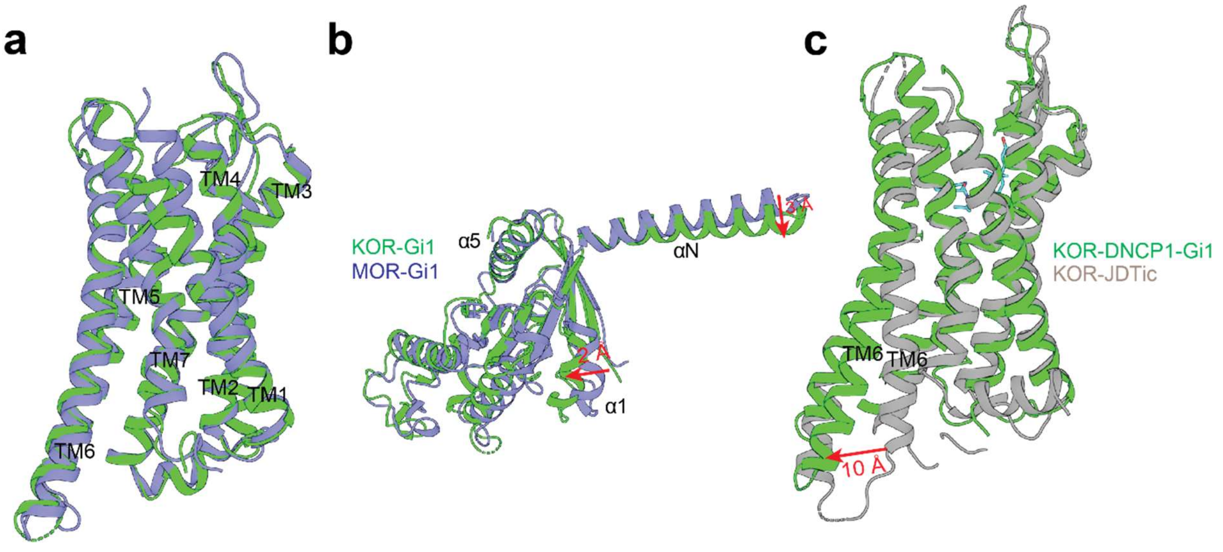

Supplementary Fig. 18 | Overlay of KOR-G$_{i1}$ and MOR-G$_{i1}$ complex structures. a, Overlay shows similar receptor conformation. **b,** The G$_{i1}$ in KOR-DNCP-β-NalA(**1**) and MOR-DAMGO (PDB: 6DDE) shows differences in α1 and αN helices. **c,** Alignment of active KOR-DNCP-β-NalA(**1**) and inactive KOR-JDTic (PDB: 4DJH) structures shows a 10 Å outward movement of TM6.

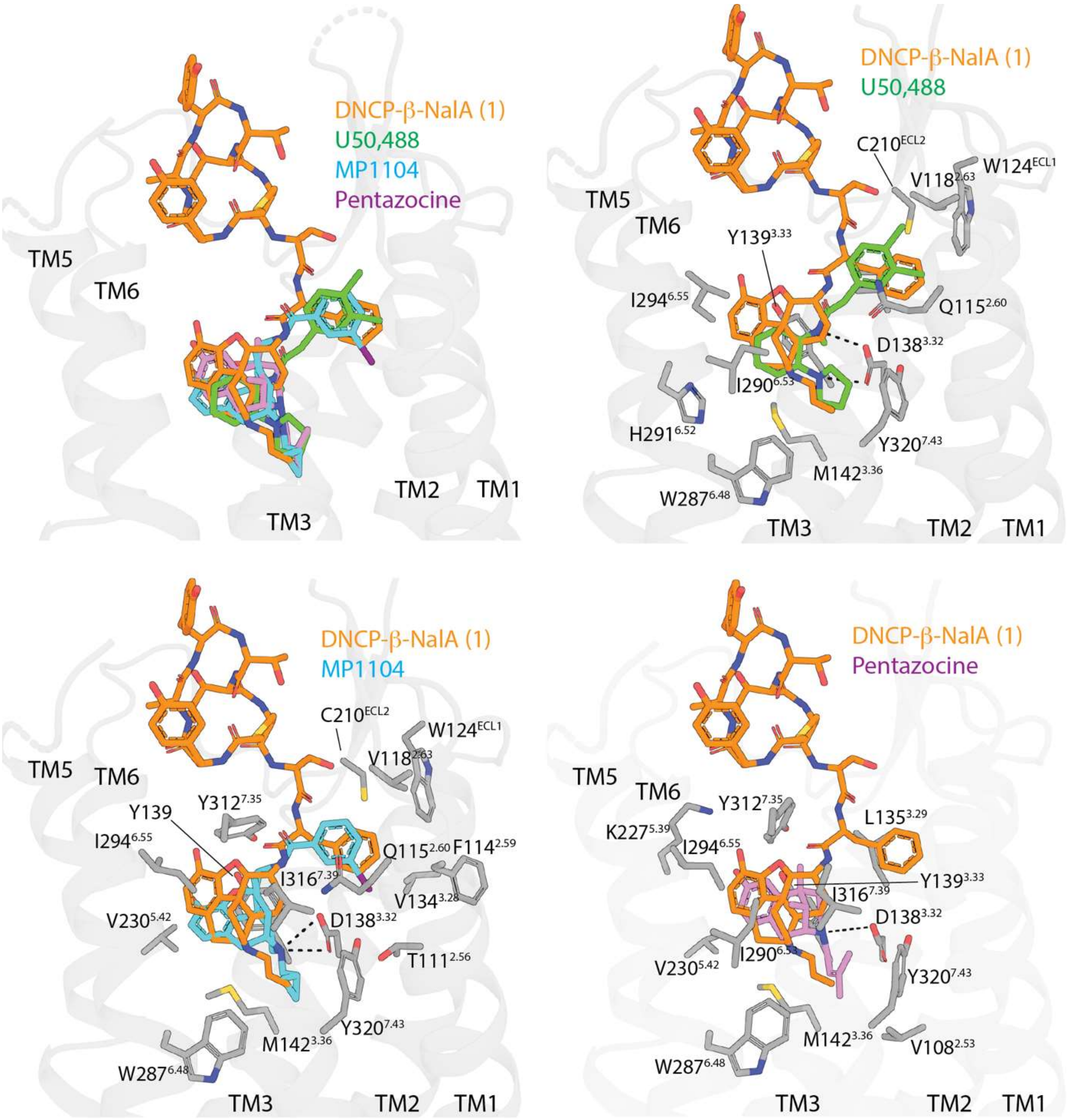

Supplementary Fig. 19 | Alignment of DNCP-β-NalA(1), U50,488, MP1104 and pentazocine. U50,488 and pentazocine were docked into the KOR-DNCP-β-NalA(**1**) structure using Maestro Schrodinger software. The binding pose of MP1104 was from the previously crystal structure (PDB: 6B73). Residues within 4 Å of each ligand are shown for U50,488, MP1104, and pentazocine, respectively.

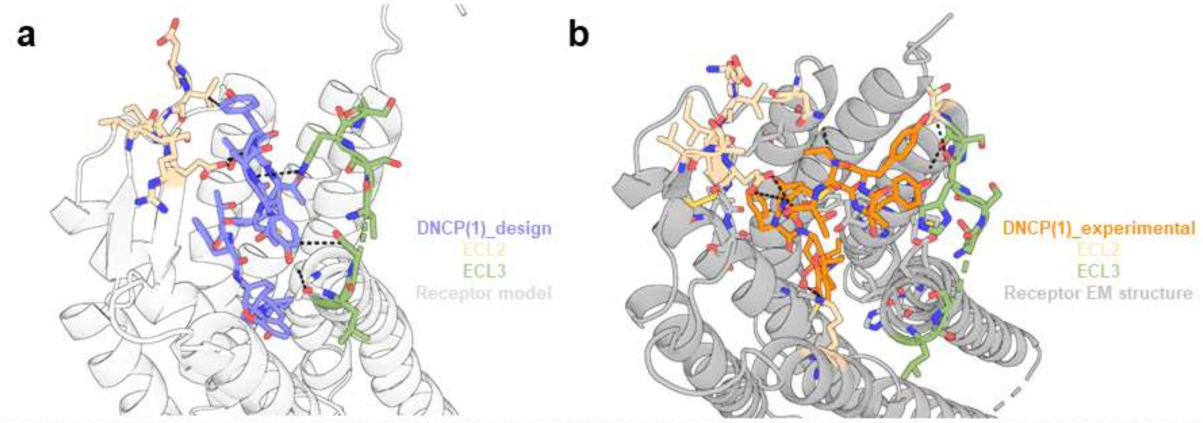

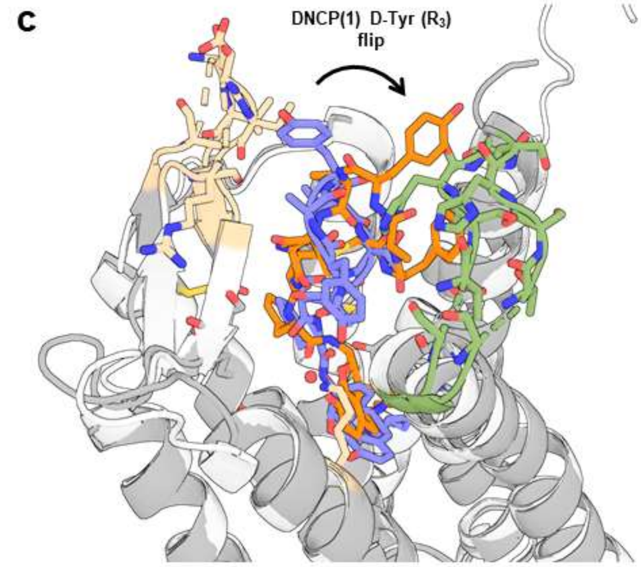

d

Key Interactions in ECL2 and ECL3

Design			Experimental		
KOR	Ligand	Region	KOR	Ligand	Region
$E209^{ECL2}$	R_4 (D-Thr)	ECL2	$E209^{ECL2}$	R_4 (D-Thr)	ECL2
$C210^{ECL2}$	R_7 (Thr)		$C210^{ECL2}$	R_7 (Thr)	
$S211^{ECL2}$	R_7 (Thr)		$S211^{ECL2}$	R_7 (Thr)	
$R202^{ECL2}$<br>$D206^{ECL2}$<br>$V207^{ECL2}$	R_3 (D-Tyr)	ECL2	$H304^{ECL3}$<br>$S305^{7.28}$<br>$T306^{7.29}$	R_3 (D-Tyr)	ECL3
$E297^{6.58}$<br>$A298^{6.59}$<br>$G300^{6.61}$<br>$H304^{ECL3}$	R_1 (Tyr)	ECL3	$S303^{ECL3}$<br>$H304^{ECL3}$<br>$S305^{7.28}$<br>$A308^{7.31}$<br>$L309^{7.32}$	R_1 (Tyr)	ECL3

Supplementary Fig. 20 | Comparison of computationally designed peptide and experimental cryo-EM structure of kappa-opioid receptor bound to DNCP-β-NalA(1). a, Shows the original designed model of DNCP-β-NalA(**1**), highlighting interactions between the peptide and ECL2/3 of the kappa-opioid receptor. **b,** Shows the experimentally determined structure of DNCP-β-NalA(**1**), highlighting interactions between the peptide and the ECL2/3 of the kappa-opioid receptor. **c,** Shows an overlay of the two structures (model and experimental). **d,** Table of interactions between the ligand and the ECL2/ECL3 of the kappa-opioid receptor – key difference seen between the computational design and experimental structure in the ECL2 interactions. Ballesteros-Weinstein residue numbers are according to https://gpcrdb.org/.

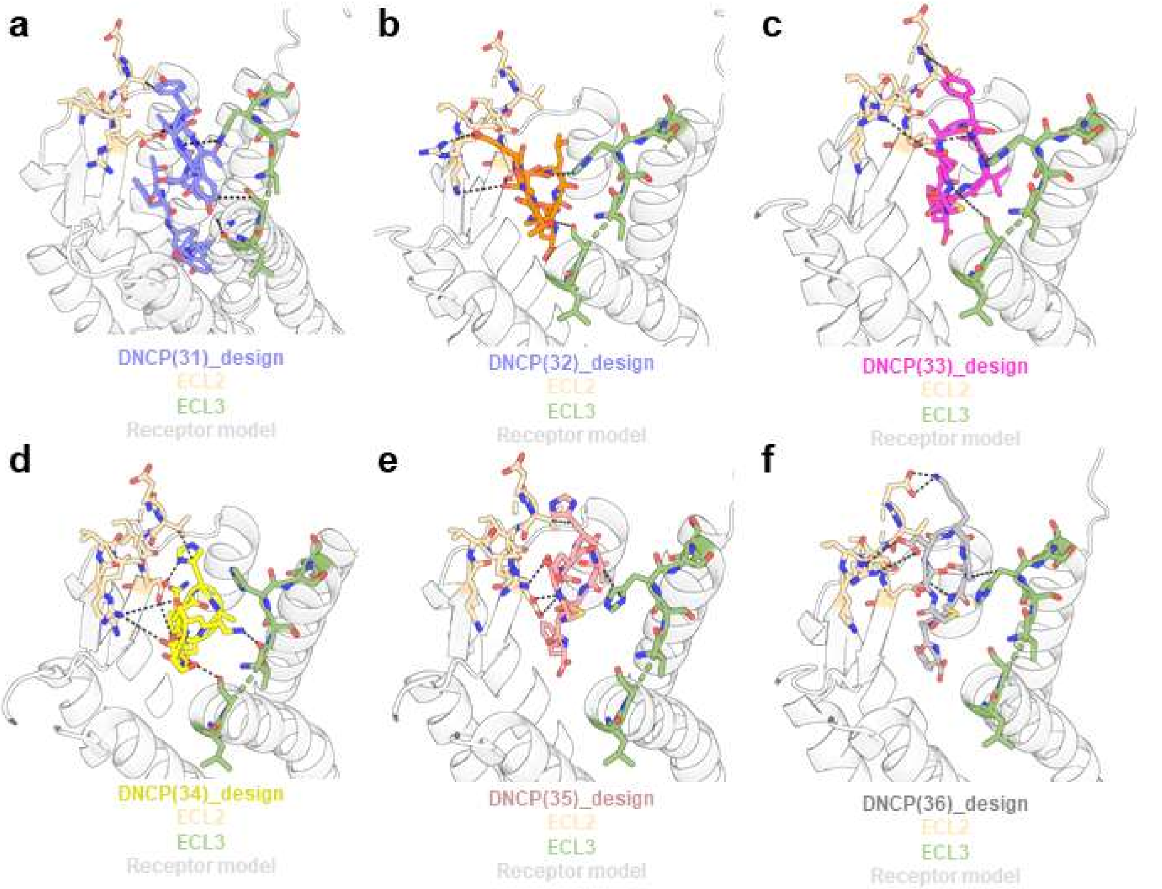

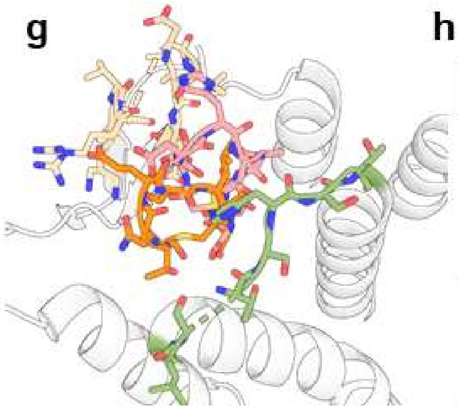

h

Key Interactions in the ECL2 and ECL3 for the designs							
KOR Residue	KOR Residue	Design Interaction (Yes/No)					
		DNCP 31	DNCP 32	DNCP 33	DNCP 34	DNCP 35	DNCP 36
ECL2	E209^{ECL2}	Yes	Yes	Yes	Yes	Yes	Yes
	S211^{ECL2}	Yes	Yes	No	Yes	No	No
	R202^{ECL2}	Yes	Yes	Yes	No	Yes	Yes
	D206^{ECL2}	Yes	No	Yes	Yes	Yes	Yes
	V207^{ECL2}	Yes	Yes	Yes	Yes	Yes	Yes
ECL3	E297$^{6.58}$	Yes	Yes	Yes	Yes	No	No
	A298$^{6.59}$	Yes	Yes	No	No	No	No
	G300$^{6.61}$	Yes	Yes	No	Yes	No	No
	H304^{ECL3}	Yes	Yes	Yes	Yes	Yes	Yes

Supplementary Fig. 21 | Annotation of interactions between computationally designed peptides and the kappa-opioid receptor. Highlighting interactions between kappa-opioid receptor model and **a,** DNCP 31 design, **b,** DNCP 32 design, **c,** DNCP 33 design, **d,** DNCP 34 design, **e,** DNCP 35 design and **f,** DNCP 36 design. **g,** Shows the overlay of DNCP 32 and DNCP 35 showing diversity in binding pocket geometry. **h,** Highlights interactions found across DNCP 31-36 designs.

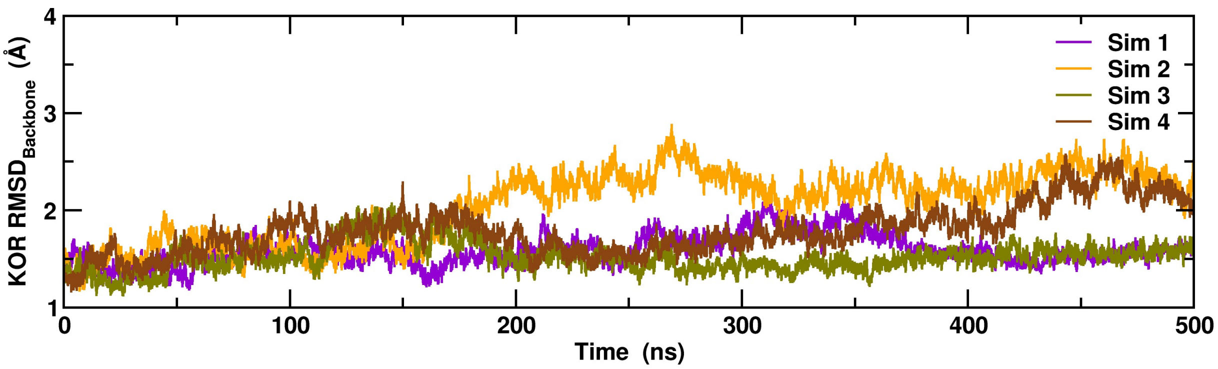

Supplementary Fig. 22 | Time course plots for the root mean square deviation (RMSD) for the protein backbone atoms during molecular dynamics simulations

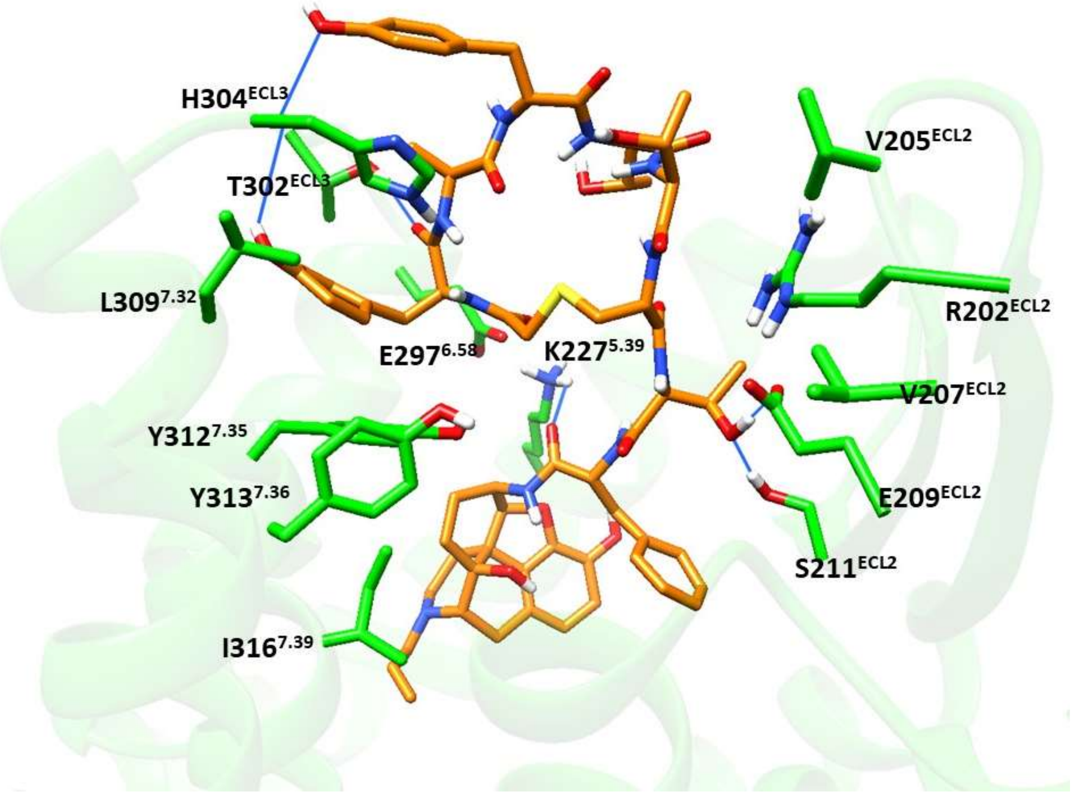

Supplementary Fig. 23 | Representative snapshot from the molecular dynamics simulations showing detailed interactions between KOR (green) and the peptide ring of DNCP-β-NalA(1) (orange). Residues are mainly located in TM2, TM6, TM7, ECL2 and ECL3. The blue lines illustrate H-bonds interactions.

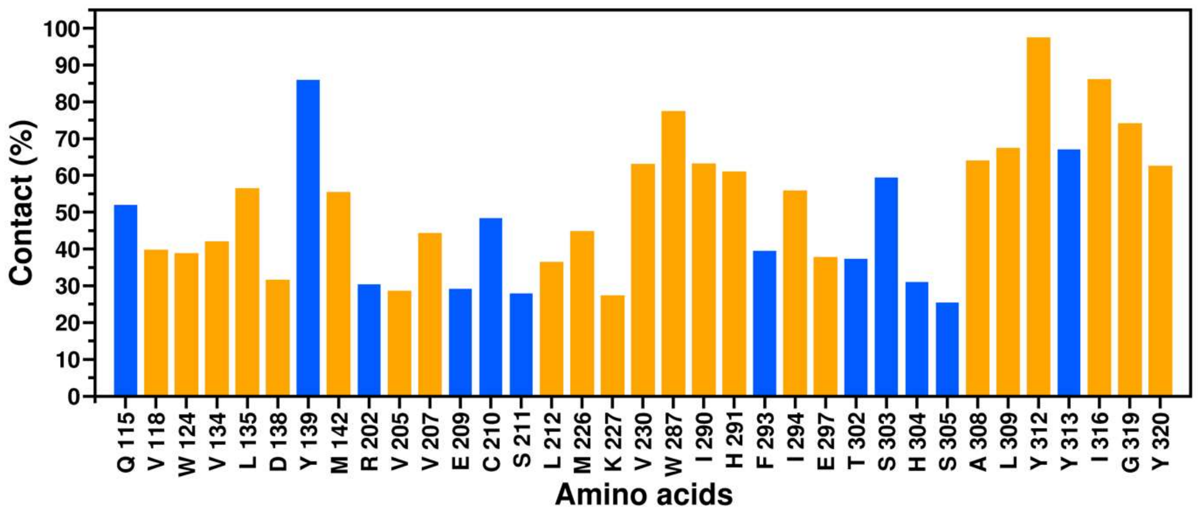

Supplementary Fig. 24 | Fraction of contact observed in the combined trajectories of molecular dynamics simulations (2 μs total). A 4.5 Å cut-off distance between all heavy atoms of DNCP-β-NalA(**1**) and KOR. Orange bars represent hydrophobic interactions and blue bars represent hydrogen bonding interactions. For clarity of visualization, fraction of contacts less than 25% are hidden.

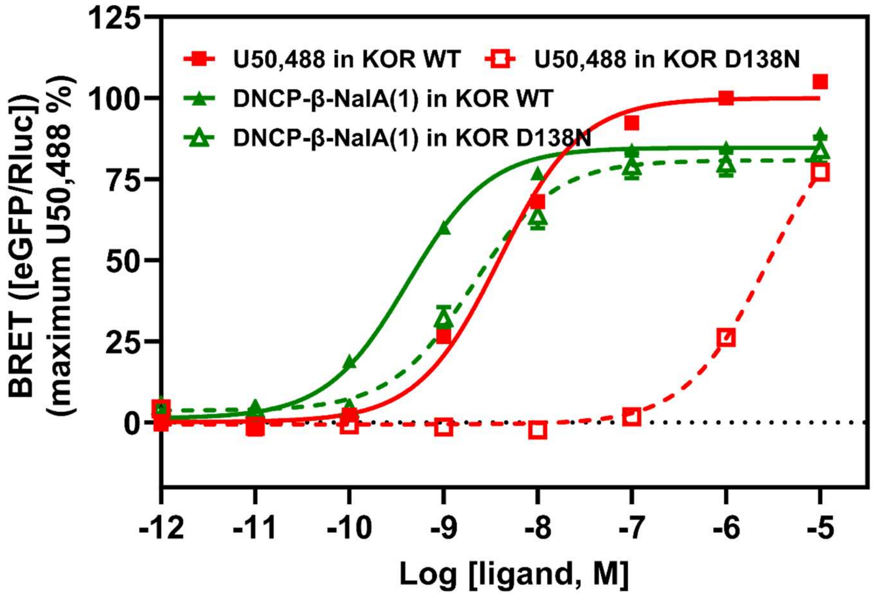

Supplementary Fig. 25 | The effect of D138$^{3.32}$N mutation on DNCP-β-NalA(1)-mediated G_{i1} activation. The assays were conducted in HEK 293T cells transiently transfected with KOR WT or D138$^{3.32}$N mutant using BRET2-G protein activation. Data are presented as the mean ± s.e.m. from three independent experiments. The KOR agonist U50,488 was used as a reference.

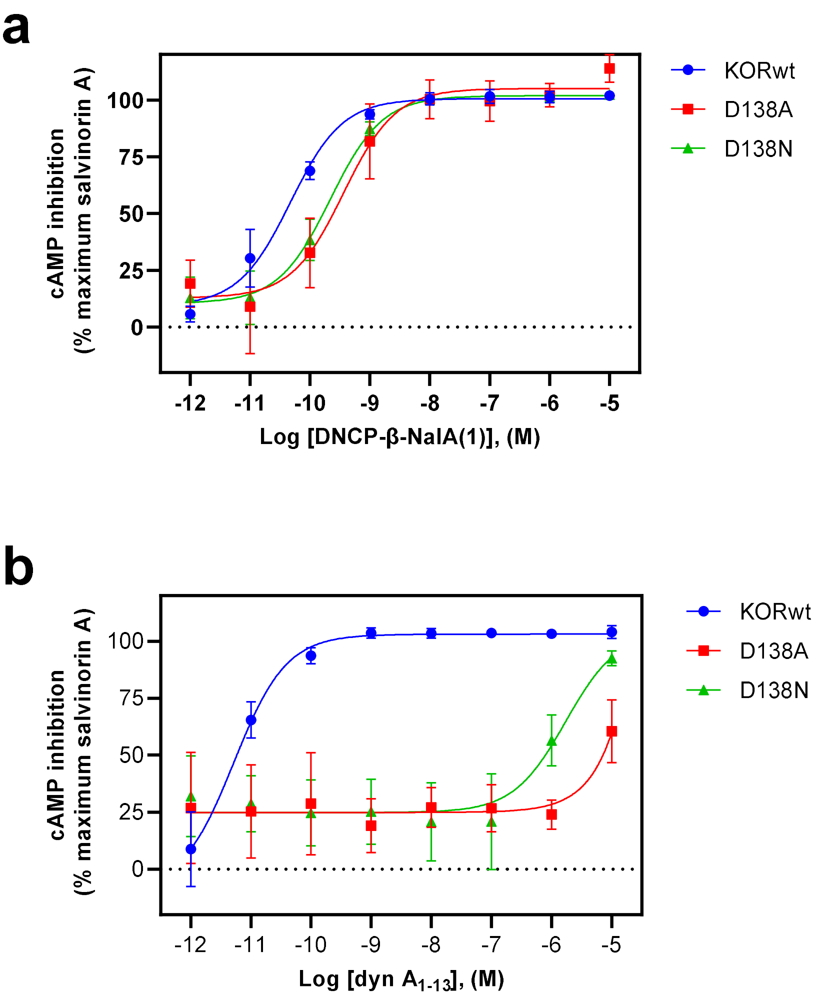

Supplementary Fig. 26 | The effects of KOR D138A or D138N on *DN*CP-β-NalA(1) or dyn A_{1-13} revealed by cAMP inhibition assays. a, DNCP-β-NalA(**1**) and **b**, dyn A_{1-13} dyn. Salvinorin A was used as a reference ligand to normalize the data. Data are presented as the mean ± s.e.m. from three independent experiments. See **Supplementary Table 9**.

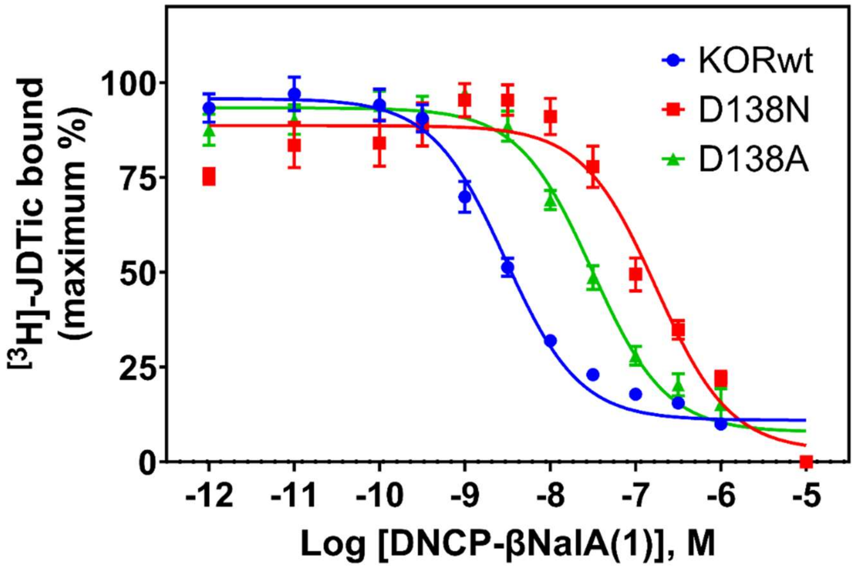

Supplementary Fig. 27 | Measurement of binding affinity in KOR D138$^{3.32}$A or D138$^{3.32}$N mutants by radioligand binding assays. The antagonist [^{3}H]JDTic was used as the radioligand. Data are presented as the mean ± s.e.m. from three independent experiments. See Supplementary Table 10.

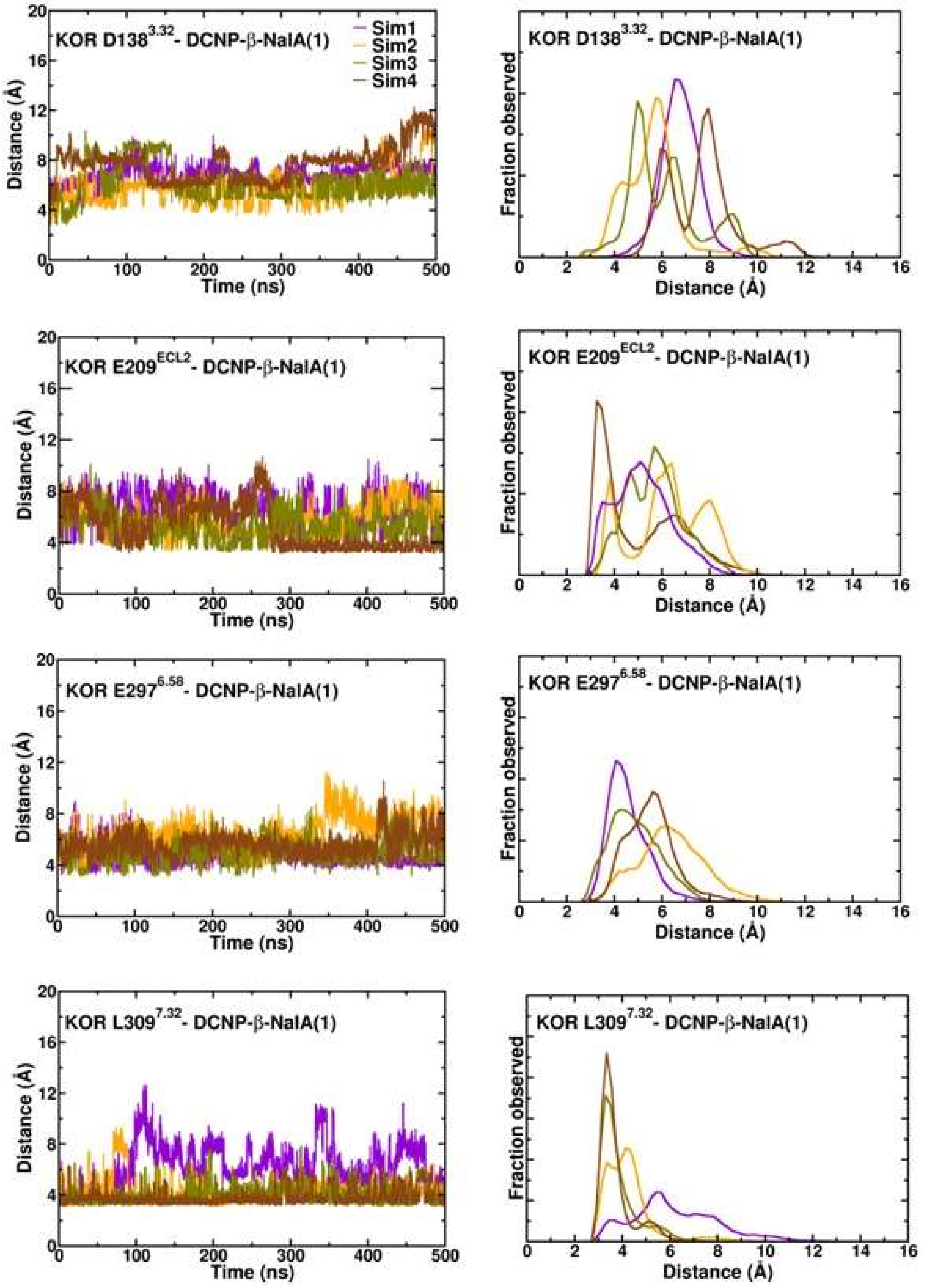

Supplementary Fig. 28 | Time course of the distance between the ligand and protein amino acid residue (right) and corresponding probability distribution (left)

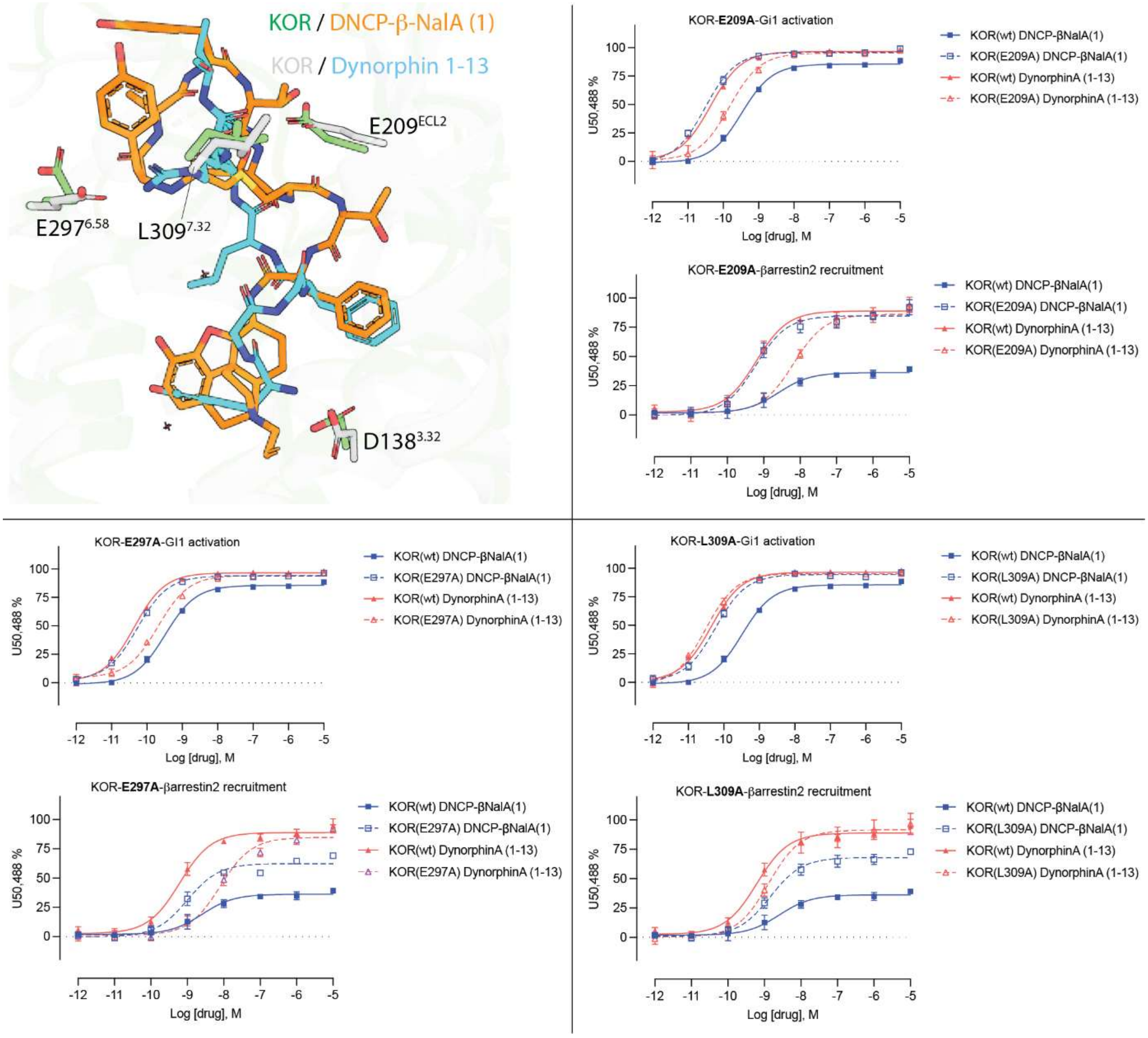

Supplementary Fig. 29 | Comparison of the binding poses between DNCP-β-NalA(1) and dynorphin $A_{1\text{-}13}$. DNCP-β-NalA(**1**) (orange) shares parts of the binding pocket with dynA (cyan). Functional characterization of binding pocket residues shows that they have distinct effects on the potency and/or efficacy of ligand-mediated G protein or arrestin activation. Data are presented as the mean ± s.e.m. from three independent experiments. See Supplementary Table 13 and 14.

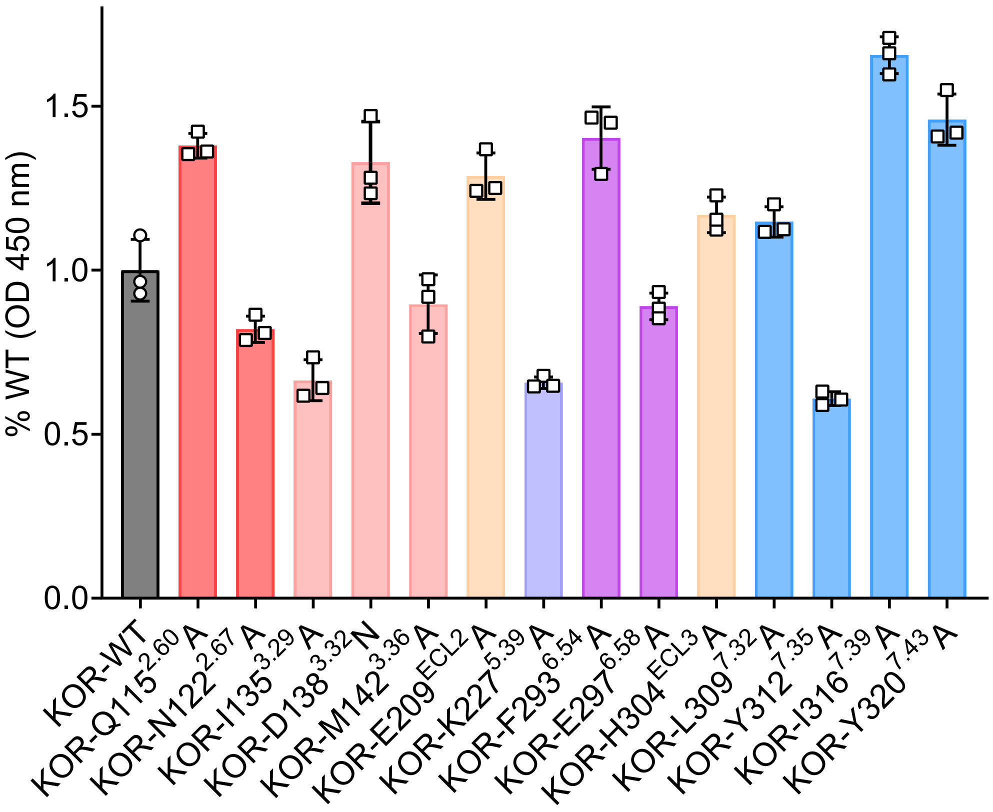

Supplementary Fig. 30 | Cell surface expression of KOR binding pocket mutants by ELISA. KOR WT or mutants were transiently transfected into HEK 293 cells, and the cell surface expression was examined after 48 h by ELISA. Data are presented as the mean ± s.e.m. from three independent experiments, normalized to 100% (KOR-WT) expressed as 1.0.

Supplementary Table 1 | Standard Operating Procedure for Liberty Blue™ automated microwave peptide synthesizer used to produce peptide precursors

Entry	Operation[a]	Solvent/reagents	Temp.	Time (repetitions)
1	Resin transfer[b,c]	DMF	25°C	2 min (2×)
2	Resin swelling[b]	DMF	25°C	5 min (1×)
3	Fmoc deprotection	20% piperidine in DMF + Oxymapure (0.1 M, 4 mL)	90°C[d]	1 min (1×)
4	Washing[e]	DMF	25°C	15 s (4×)
5	Coupling	Fmoc-AA in DMF (0.2 M, 5 eq, 2.5 mL) + Activator: DIC in DMF (1 M, 1 mL) + Base: OxymaPure in DMF (1 M, 0.5 mL)	90°C[d]	4 min (1×)
6	Washing[e]	DMF	25°C	15 s (2×)
7	Final Fmoc deprotection[f]	20% piperidine in DMF + Oxymapure (0.1 M, 4 mL)	90°C[d]	1 min (1×)
8	Washing[e,f]	DMF	25°C	15 s (5×)

[a] After each operation, the reaction vessel is drained
[b] These operations are performed only for the first cycle during SPPS
[c] For each transfer of resin from resin loader (HT-12) to the reaction vessel, DMF (15 mL) was used; followed by draining the reaction vessel (5 s)
[d] Microwave heating
[e] Washing involves addition of DMF (5 s), N_2 bubbling (5 s) and draining the reaction vessel (5 s)
[f] These operations are performed only after complete peptide is assembled.
AA = amino acid

Supplementary Table 2 | Mass spectrometry and HPLC data of peptide ligands and DNCP-β-NalA conjugates

Peptide	HPLC purity (%)	HPLC retention times (min)	$[M+H]^+$ (calculated)	$[M+H]^+$ (observed)
**DNLPs**				
**11**	99.5	5.8	968.4	968.4
**12**	98.9	5.7	899.4	899.4
**13**	94.0	6.6	888.4	888.4
**14**	97.5	3.9	836.3	836.3
**15**	98.1	3.3	890.4	890.4
**16**	97.5	4.1	811.4	811.3
**DNCPs with amidated *C*-term**				
**21**	97.3	6.5	1008.4	1008.3
**22**	95.7	6.8	939.4	939.4
**23**	97.7	6.8	928.4	928.4
**24**	96.3	4.2	876.3	876.3
**25**	95.6	3.6	930.4	930.4
**26**	99.3	4.3	851.4	851.3
**DNCPs with free *C*-term for conjugation with β-NalA**				
**31**	98.5	7.8	1009.4	1009.4
**32**	95.4	7.1	940.4	940.3
**33**	99.4	8.1	929.4	929.3
**34**	98.4	6.8	877.3	877.3
**35**	99.6	5.1	931.4	931.4
**36**	99.3	4.9	852.3	852.3
**DNCP-β-NalA**				
**1**	99.9	33.0	1319.565	1319.569
**2**	99.0	33.1	1250.539	1250.543
**3**	98.0	36.8	1239.539	1239.557
**4**	97.0	26.9	1187.482	1187.484

Analysis of *de novo* linear peptides (DNLP) and de novo cyclic peptides (DNCP) and the DNCP-β-NalA conjugates. Purity of DNLPs and DNCPs (calculated by automatic peak integration from 5-15 min) was determined by analytical RP-UPLC using a Phenomenex Luna Omega C_{18} column (1.6 µm, 100 Å, 50 x 2.1 mm) with a linear gradient of 1-61% of solvent B (90% acetonitrile, 10% H_2O, 0.05% trifluoroacetic acid) in 15 min and a flowrate of 0.6 mL min^{-1}. Purity of DNCP-β-NalA conjugates (calculated by automatic peak integration from 5-45 min) was assessed by RP-HPLC using a Phenomenex Jupiter C_{18} column (5 µM, 300 Å, 50 × 2 mm) and a linear gradient of 5-65% solvent B (90% acetonitrile, 10% H_2O, 0.1% trifluoroacetic acid) in 60 min at a flow rate of 1 mL min^{-1}. Monoisotopic masses ($[M+H]^+$) of DNLPs and DNCPs were identified by low-resolution ESI-MS on a LCMS-2020 and of DNCP-β-NalA conjugates by high-resolution ESI-MS on a LC-QqTOF compact. Calculated mass errors (in ppm) for DNCP-β-NalA(**1**), DNCP-β-NalA(**2**), DNCP-β-NalA(**3**) and DNCP-β-NalA(**4**) were 3, 3, 15 and 2, respectively.

Supplementary Table 3 | In vitro pharmacological properties of DNCP-β-NalA conjugates at the KOR

Ligand	Radioligand binding assay (mouse KOR)	Potency/efficacy cAMP assay (mKOR)		Potency/efficacy β-arrestin-2 recruitment assay (mKOR)		Potency/efficacy β-arrestin-1 recruitment assay (human KOR)	
	**K_i (M)**	**EC_{50} (M)**	**E_{max} (%)**	**EC_{50} (M)**	**E_{max} (%)**	**EC_{50} (M)**	**E_{max} (%)**
dynA$_{1\text{-}13}$	-	4.9 ± 1.8	100	3.1. ± 0.6 x 10^{-8}	100	-	-
U50,488	7.7 ± 0.4 x 10^{-9}	2.2 ± 0.1 x 10^{-9}	100	-	-	3.3 ± 0.8 x 10^{-7}	100
U69,593	-	-	-	5.0 ± 1.1 x 10^{-7}	120 ± 11	-	-
MP1104	-	-	-	-	-	3.2 ± 0.9 x 10^{-9}	91 ± 8
Pentazocine	-	-	-	-	-	1.0 ± 0.3 x 10^{-6}	38 ± 24
β-NalA	7.2 ± 0.9 x 10^{-8}	1.3 ± 0.1 x 10^{-7}	61 ± 7	1.5 ± 0.2 x 10^{-8}	51 ± 5	-	-
DNCP-β-NalA(**1**)	3.9 ± 0.7 x 10^{-9}	2.0 ± 0.1 x 10^{-9}	92 ± 1	2.2 ± 1.3 x 10^{-8}	41 ± 2	2.8 ± 0.7 x 10^{-8}	30 ± 11
DNCP-β-NalA(**2**)	3.1 ± 0.4 x 10^{-8}	7.5 ± 0.6 x 10^{-9}	89 ± 2	-	-	-	-
DNCP-β-NalA(**3**)	2.4 ± 0.4 x 10^{-8}	1.4 ± 0.7 x 10^{-8}	99 ± 1	-	-	-	-
DNCP-β-NalA(**4**)	1.3 ± 0.1 x 10^{-8}	1.0 ± 0.1 x 10^{-9}	101 ± 2	-	-	-	-

Percentage stimulation (E_{max}, %) relative to the reference full agonist U50,488 or dynA$_{1\text{-}13}$; data are from three independent experiments; values represent means ± s.e.m.

Supplementary Table 4 | Agonist potencies and efficacies of DNCP-β-NalA(1) and reference ligands at the human KOR, MOR and DOR in the [^{35}S]GTPγS binding assay

Ligand	KOR[a]		MOR[a]		DOR[a]	
	EC_{50} (M)	E_{max} (%)	EC_{50} (M)	E_{max} (%)	EC_{50} (M)	E_{max} (%)
DNCP-β-NalA(**1**)	$5.5 \pm 1.7 \times 10^{-9}$	83 ± 4	-[b]		$3.1 \pm 1.5 \times 10^{-7}$	31 ± 3
β-NalA	$1.5 \pm 0.3 \times 10^{-7}$	22 ± 3	-[b]		-[b]	
U69,593	$2.9 \pm 0.9 \times 10^{-8}$	100				
$dynA_{1-13}$	$2.7 \pm 0.9 \times 10^{-9}$	100				
DAMGO			$1.8 \pm 0.6 \times 10^{-8}$	100		
DPDPE					$1.2 \pm 0.1 \times 10^{-8}$	100

[a] Determined in the [^{35}S]GTPγS binding assays using membranes from CHO cells stably expressing the human opioid receptors; percentage stimulation (E_{max}, %) relative to the reference full agonist U69,593 (KOR), $dynA_{1-13}$ (KOR), DAMGO (MOR) and DPDPE (DOR).

[b] - denotes no stimulation up to 10 μM.

values represent means ± s.e.m. from three independent experiments.

Supplementary Table 5 | TRUPATH potency data of DNCP-β-NalA(1) and reference KOR agonists at the human KOR

**Gα$_{i/o}$ subtype**	**Gα$_{i1}$**	**Gα$_{i2}$**	**Gα$_{i3}$**	**Gα$_{oA}$**	**Gα$_{oB}$**	**Gα$_{gastducin}$**	**Gα$_{z}$**
**Ligand**	**EC$_{50}$ (M)**	**EC$_{50}$ (M)**	**EC$_{50}$ (M)**	**EC$_{50}$ (M)**	**EC$_{50}$ (M)**	**EC$_{50}$ (M)**	**EC$_{50}$ (M)**
U50,488	3.7 ± 1.0 x 10^{-9}	1.4 ± 0.4 x 10^{-9}	3.6 ± 0.8 x 10^{-9}	4.9 ± 1.1 x 10^{-9}	5.5 ± 1.0 x 10^{-9}	1.4 ± 0.3 x 10^{-8}	0.9 ± 0.4 x 10^{-9}
MP1104	0.1 ± 0.0 x 10^{-9}	0.3 ± 0.1 x 10^{-9}	0.2 ± 0.1 x 10^{-9}	1.2 ± 0.6 x 10^{-9}	0.6 ± 0.2 x 10^{-9}	1.6 ± 0.2 x 10^{-9}	1.0 ± 0.2 x 10^{-11}
Pentazocine	1.7 ± 0.8 x 10^{-8}	1.4 ± 0.9 x 10^{-8}	3.3 ± 1.0 x 10^{-8}	3.3 ± 1.1 x 10^{-9}	9.3 ± 5.7 x 10^{-9}	1.2 ± 0.8 x 10^{-8}	3.3 ± 1.0 x 10^{-8}
DNCP-β-NalA(**1**)	1.7 ± 0.5 x 10^{-9}	0.9 ± 0.4 x 10^{-9}	0.7 ± 0.2 x 10^{-9}	3.1 ± 1.5 x 10^{-8}	2.4 ± 0.9 x 10^{-9}	0.1 ± 0.0 x 10^{-9}	1.4 ± 0.3 x 10^{-9}

Data are from four independent experiments; values represent means ± s.e.m.

Supplementary Table 6 | TRUPATH efficacy data of DNCP-β-NalA(1) and reference KOR agonists at the human KOR

**$G\alpha_{i/o}$ subtype**	**$G\alpha_{i1}$**	**$G\alpha_{i2}$**	**$G\alpha_{i3}$**	**$G\alpha_{oA}$**	**$G\alpha_{oB}$**	**$G\alpha_{gastducin}$**	**$G\alpha_{z}$**
**Ligand**	**E_{max} (%)**	**E_{max} (%)**	**E_{max} (%)**	**E_{max} (%)**	**E_{max} (%)**	**E_{max} (%)**	**E_{max} (%)**
U50,488	100 ± 4	100 ± 4	100 ± 3	100 ± 5	100 ± 6	100 ± 5	100 ± 4
MP1104	100 ± 4	65 ± 6	85 ± 3	87 ± 7	101 ± 8	95 ± 5	101 ± 3
Pentazocine	58 ± 4	42 ± 6	54 ± 3	35 ± 8	45 ± 7	79 ± 6	40 ± 2
DNCP-β-NalA(**1**)	81 ± 4	62 ± 6	77 ± 3	56 ± 7	76 ± 6	101 ± 5	48 ± 2

Data are from four independent experiments; values represent means ± s.e.m.

Supplementary Table 7 | Structural parameters of KOR-G_{i1}-DNCP-β-NalA(1) parameters

	KOR-G_{i1}-DNCP-β-NalA(**1**)<br>(EMD-29026)<br>(PDB: 8FEG)
**Data collection and processing**	
Magnification	45,000
Voltage (kV)	200
Electron exposure (e-/Å^2)	49
Number of movies used	4027
Defocus mean (SD) µm	1.5 (0.4)
Pixel size (Å)	0.88
Symmetry imposed	C1
Initial particle images (No.)	1,742,555
Final particle images (No.)	491,092
Map resolution (Å)	2.54
FSC threshold 0.143	0.143
Map resolution range (Å)	2.3-5.8
**Refinement**	
Initial model used (PDB code)	6DDE
Model resolution (Å)	2.89
FSC threshold	0.5
Map sharpening B factor (Å^2)	-83.4
Model composition	
Non-hydrogen atoms	8853
Protein residues	1120
Ligands	1
B factor (Å^2)	
Protein	58.65
Ligands	69.97
Water	62.68
R.m.s. deviations	
Bond lengths (Å)	0.005
Bond angles (°)	0.841
Validation	
MolProbity score	1.56
Clashscore	5.01
Poor rotamers (%)	0
Ramachandran plot	
Favored (%)	95.72
Allowed (%)	4.28
Disallowed (%)	0

Supplementary Table 8 | The average distance between Cα-atoms of kappa-opioid receptor and Cα-atoms of cyclic-peptide of DNCP-β-NalA(1)

KOR	Ligand	Region	Distance (Cryo-EM)	Distance (Simulations)
E209 ECL2	R4 (D-Thr)	ECL2	8.89	10.99 ± 1.66
C210 ECL2	R7 (Thr)	ECL2	5.03	7.33 ± 1.50
S211 ECL2	R7 (Thr)	ECL2	4.63	6.91 ± 0.48
H304 ECL3	R3 (D-Tyr)	ECL3	6.81	7.39 ± 1.38
S305 ECL3	R3 (D-Tyr)	ECL3	8.35	9.95 ± 1.24
T306 ECL3	R3 (D-Tyr)	ECL3	8.06	11.01 ± 1.64
E297	R1 (Tyr)	ECL3	6.81	7.66 ± 0.67
A298	R1 (Tyr)	ECL3	8.96	10.49 ± 0.82
G300	R1 (Tyr)	ECL3	11.38	11.72 ± 0.85
H304 ECL3	R1 (Tyr)	ECL3	9.47	8.74 ± 0.76
S303 ECL3	R1 (Tyr)	ECL3	10.34	9.41 ± 1.40
S305	R1 (Tyr)	ECL3	10.75	9.93 ± 0.55
A308	R1 (Tyr)	ECL3	7.57	8.55 ± 0.59
L309	R1 (Tyr)	ECL3	5.93	6.55 ± 0.56

values represent means ± s.d. from four independent MD simulations

Supplementary Table 9 | cAMP inhibition efficacy data of DNCP-β-NalA(1) at the human KOR and D138 mutants

Receptor	DNCP-NalA(1)		Dynorphin A_{1-13}	
	**pEC_{50} (M)**	**E_{max} (%)**	**pEC_{50} (M)**	**E_{max} (%)**
KOR WT	10.3 ± 0.1	100.6 ± 0.9	11.3 ± 0.1	103.1 ± 0.9
KOR D138A	9.4 ± 0.1	105.1 ± 2.4	N.A.	N.A.
KOR D138N	9.7 ± 0.1	101.9 ± 1.0	5.8 ± 0.2	104.5 ± 8.2

Data were acquired by cAMP assays normalized to salvinorin A; values represent mean pEC_{50} ± s.e.m. from three independent replicates; N.A. represents no activity

Supplementary Table 10 | Binding data of DNCP-β-NalA(1) at the human KOR and D138 mutants

Receptor	pK_i (M)
KOR WT	8.87 ± 0.05
KOR D138A	7.85 ± 0.06
KOR D138N	7.10± 0.09

Data were acquired by radioligand competitive binding assay using [^{3}H]JDTic (see **Supplementary Fig. 27**); values represent mean pK_i ± s.e.m. from three independent replicates

Supplementary Table 11 | Biosensor efficacy data of DNCP-β-NalA(1) and U50,488 at the human KOR and mutants

	DNCP-β-NalA(1)				U50,488	
Receptor	$G\alpha_i$		β-arrestin-2		$G\alpha_i$	
	pEC_{50} (M)	E_{max} (%)	pEC_{50} (M)	E_{max} (%)	pEC_{50} (M)	E_{max} (%)
KOR WT	9.51 ± 0.06	85.87 ± 1.20	8.36 ± 0.11	35.34 ± 1.13	8.41 ± 0.03	99.95 ± 1.02
KOR Q115A	9.68 ± 0.07	90.23 ± 1.46	8.47 ± 0.11	60.05 ± 1.71		
KOR I135A	9.11 ± 0.16	45.75 ± 1.95	N.A.	N.A.		
KOR D138N	8.77 ± 0.04	89.58 ± 0.98			5.56 ± 0.06	98.56 ± 4.31
KOR M142A	7.39 ± 0.08	69.43 ± 1.97	5.64 ± 0.21	91.66 ± 11.62		
KOR K227A	8.75 ± 0.09	47.88 ± 1.19	7.98 ± 0.50	16.89 ± 2.26		
KOR I316A	8.65 ± 0.03	85.75 ± 0.72	7.40 ± 0.22	22.7 ± 1.59		
KOR Y320A	7.98 ± 0.07	86.84 ± 2.05	6.46 ± 0.14	83.47 ± 4.81		

Data were acquired by BRET biosensor assays; values represent mean pEC50 ± s.e.m. from three independent replicates; N.A. represents no activity

Supplementary Table 12 | Biosensor efficacy data of DNCP-β-NalA(1) at the human KOR and mutants

Receptor	$G\alpha_i$		β-arrestin-2	
	pEC_{50} (M)	E_{max} (%)	pEC_{50} (M)	E_{max} (%)
KOR WT	9.58 ± 0.04	86.51 ± 0.80	8.50 ± 0.13	29.77 ± 0.99
KOR E209A	10.64 ± 0.03	99.31 ± 0.52	9.14 ± 0.06	87.35 ± 1.31
KOR F293A	8.80 ± 0.03	85.55 ± 0.78	7.30 ± 0.12	26.48 ± 1.01
KOR E297A	10.25 ± 0.03	96.28 ± 0.53	8.99 ± 0.08	64.57 ± 1.35
KOR H304A	9.74 ± 0.02	92.57 ± 0.43	8.37 ± 0.12	34.48 ± 1.18
KOR L309A	10.27 ± 0.02	95.98 ± 0.44	8.88 ± 0.06	62.23 ± 1.08
KOR Y312A	9.01 ± 0.03	82.46 ± 0.65	N.A	N.A

Data were acquired by BRET biosensor assays; values represent mean pEC_{50} ± s.e.m. from three independent replicates; N.A. represents no activity

Supplementary Table 13 | G_i efficacy data of DNCP-β-NalA(1) at the human KOR and mutants

Receptor	DNCP-NalA(1)		Dynorphin A_{1-13}	
	pEC$_{50}$ (M)	E_{max} (%)	pEC$_{50}$ (M)	E_{max} (%)
KOR WT	9.48 ± 0.02	85.54 ± 0.48	10.36 ± 0.03	96.59 ± 0.52
KOR E209A	10.52 ± 0.03	95.82 ± 0.52	9.83 ± 0.04	95.27 ± 0.83
KOR E297A	10.27 ± 0.02	94.03 ± 0.37	9.69 ± 0.02	94.35 ± 0.52
KOR L309A	10.24 ± 0.02	94.59 ± 0.50	10.52 ± 0.02	95.45 ± 0.46

Data were acquired by G_i biosensor assays (see **Supplementary Fig. 28**); values represent mean pEC$_{50}$ ± s.e.m. from three independent replicates; N.A. represents no activity

Supplementary Table 14 | β-arrestin-2 efficacy data of *DN*CP-β-NalA(1) at the human KOR and mutants

Receptor	*DN*CP-NalA(1)		Dynorphin $A_{1\text{-}13}$	
	**pEC_{50} (M)**	**E_{max} (%)**	**pEC_{50} (M)**	**E_{max} (%)**
KOR WT	8.60 ± 0.09	36.22 ± 0.90	9.21 ± 0.04	88.90 ± 1.04
KOR E209A	9.22 ± 0.06	84.68 ± 1.32	8.18 ± 0.04	86.04 ± 1.12
KOR E297A	9.00 ± 0.06	62.44 ± 1.05	8.10 ± 0.05	84.76 ± 1.43
KOR L309A	8.84 ± 0.05	68.01 ± 0.94	8.88 ± 0.06	91.58 ± 1.57

Data were acquired by β-arrestin-2 biosensor assays (see **Supplementary Fig. 28**); values represent mean pEC_{50} ± s.e.m. from three independent replicates; N.A. represents no activity

Supplementary Table 15 | Description of the system setup in the molecular dynamics simulations.

System Compositions	Number
kappa-opioid receptor + G proteins ($G\alpha_{i1}/G\beta_1/G\gamma_2$)	1
DNCP-β-NalA(**1**)	1
DPPC	330
DOPC	90
Cholesterol	180
Water	63573
Sodium ions	172
Chloride ions	172
Total number of atoms	70074
Simulations box type	Rectangular
Box dimension - Sim 1	118.802 119.068 188.013
Box dimension - Sim 2	119.572 119.840 185.458
Box dimension - Sim 3	119.530 119.797 185.334
Box dimension - Sim 4	119.305 119.572 186.281
