# Supplementary material for: Design and structural validation of peptide–drug conjugate ligands of the kappa-opioid receptor
Source: Nat Commun. 2023 Dec 6;14:8064. doi: 10.1038/s41467-023-43718-w (PMC10698194; doi:10.1038/s41467-023-43718-w)
Supplement: Supplementary file 3 — Description of Additional Supplementary Files [file 41467_2023_43718_MOESM3_ESM.pdf]

File Name: Supplementary Data 1

Description: Backbone generation protocol, <thioether_backbone.py>

File Name: Supplementary Data 2

Description: Backbone to anchor docking protocol, <docking_protocol.py>

File Name: Supplementary Data 3

Description: Side chain rotamer design and filtering protocol, <kappa_peptide_design.xml>

File Name: Supplementary Data 4

Description: Residue and parameter files for scripts supplied in Supplementary Data 1 - 3, i.e. <CYY.params>, <CVV.params>, <l_res.txt>, <d_res.txt>
