# Supplementary material for: Design and structural validation of peptide–drug conjugate ligands of the kappa-opioid receptor
Source: Nat Commun. 2023 Dec 6;14:8064. doi: 10.1038/s41467-023-43718-w (PMC10698194; doi:10.1038/s41467-023-43718-w)
Supplement: Supplementary file 8 — Reporting Summary [file 41467_2023_43718_MOESM8_ESM.pdf]

nature portfolio

Corresponding author(s): Gruber CW, Che T, Baker D

Last updated by author(s): Nov 7, 2023

Reporting Summary

Nature Portfolio wishes to improve the reproducibility of the work that we publish. This form provides structure for consistency and transparency in reporting. For further information on Nature Portfolio policies, see our Editorial Policies and the Editorial Policy Checklist.

Statistics

For all statistical analyses, confirm that the following items are present in the figure legend, table legend, main text, or Methods section.

n/a	Confirmed	
☐	☒	The exact sample size (n) for each experimental group/condition, given as a discrete number and unit of measurement
☐	☒	A statement on whether measurements were taken from distinct samples or whether the same sample was measured repeatedly
☐	☒	The statistical test(s) used AND whether they are one- or two-sided<br>*Only common tests should be described solely by name; describe more complex techniques in the Methods section.*
☐	☒	A description of all covariates tested
☐	☒	A description of any assumptions or corrections, such as tests of normality and adjustment for multiple comparisons
☐	☒	A full description of the statistical parameters including central tendency (e.g. means) or other basic estimates (e.g. regression coefficient) AND variation (e.g. standard deviation) or associated estimates of uncertainty (e.g. confidence intervals)
☐	☒	For null hypothesis testing, the test statistic (e.g. F, t, r) with confidence intervals, effect sizes, degrees of freedom and P value noted<br>*Give P values as exact values whenever suitable.*
☒	☐	For Bayesian analysis, information on the choice of priors and Markov chain Monte Carlo settings
☒	☐	For hierarchical and complex designs, identification of the appropriate level for tests and full reporting of outcomes
☒	☐	Estimates of effect sizes (e.g. Cohen's d, Pearson's r), indicating how they were calculated

Our web collection on statistics for biologists contains articles on many of the points above.

Software and code

Policy information about availability of computer code

Data collection	Rosetta software was applied to obtain computational design models and scores. All codes are available in the methods and supplementary data file. Cryo-EM data were collected automatically with SerialEM V3.8.2. Molecular Dynamics Simulation were performed using AMBER22, AmberTool23 and VMD.
Data analysis	Structure determination and refinement: cryoSPARC V3.1.0, Topaz 0.2.2, DeepEMhancer, UCSF Chimera 1.15, UCSF ChimeraX 1.3, Coot 0.9.5, MolProbity (built in phenix 1.18.2), Phenix 1.18.2. Python 3.0 and pandas were used to analyze scores generated in Rosetta. GraphPad Prism v.8 was used to analyze in vitro and in vivo data. PyMOL2 was used for structure visualization and image generation.

For manuscripts utilizing custom algorithms or software that are central to the research but not yet described in published literature, software must be made available to editors and reviewers. We strongly encourage code deposition in a community repository (e.g. GitHub). See the Nature Portfolio guidelines for submitting code & software for further information.

Data

Policy information about availability of data

All manuscripts must include a data availability statement. This statement should provide the following information, where applicable:
- Accession codes, unique identifiers, or web links for publicly available datasets
- A description of any restrictions on data availability
- For clinical datasets or third party data, please ensure that the statement adheres to our policy

Structural data will be available via the worldwide protein data bank (wwPDB) with accession code 8FEG; the cryoEM map of KOR-Gi1-DNCP has been deposited to

EMDB database with the accession code EMD-29026. Generated and analyzed data sets that support the findings of this study are available as Source Data and from the corresponding authors upon reasonable request.

Research involving human participants, their data, or biological material

Policy information about studies with human participants or human data. See also policy information about sex, gender (identity/presentation), and sexual orientation and race, ethnicity and racism.

Reporting on sex and gender	For the serum stability assays, venous blood was obtained from healthy volunteer without any medication prior the blood donation. Written consent was obtained prior conducting the experiment and a compensation was offered to the volunteer. Sex or gender were not considered for this study as the overall protease activity in serum of individuals between sexes are expected to be equal.
Reporting on race, ethnicity, or other socially relevant groupings	No such grouping was performed nor was it relevant for this study.
Population characteristics	Healthy male subjects were invited for a blood donation, with participants having no medications prescribed 14 days prior of experiment.
Recruitment	No special recruitment criteria were set other than stated in this point.
Ethics oversight	The study design and conduct was in accordance to the authorization of the ethical commission of the Medical University of Vienna (EK1548/2020) and with institutional guidelines.

Note that full information on the approval of the study protocol must also be provided in the manuscript.

Field-specific reporting

Please select the one below that is the best fit for your research. If you are not sure, read the appropriate sections before making your selection.

[x] Life sciences   [ ] Behavioural & social sciences   [ ] Ecological, evolutionary & environmental sciences

For a reference copy of the document with all sections, see nature.com/documents/nr-reporting-summary-flat.pdf

Life sciences study design

All studies must disclose on these points even when the disclosure is negative.

Sample size	Computational peptide design was carried in the Rosetta software suite. The Rosetta software suite is available free of charge to academic users and can be downloaded from http://www.rosettacommons.org. Raw data of score and rmsd for plots presented in the main text and supplementary information are provided in Source Data file. Instructions and inputs for running these applications, and all other data and code necessary to support the results and conclusion are provided in Source Data file. All the structures presented will be deposited in PDB. The raw in vitro and in vivo data for pharmacological assays (presented in Figures 3-5, Supplementary Figures 6, 11-14, 25-26, 28 and Supplementary Tables 3-6 and 9-14) are available as a Source Data File. HPLC traces and MS data of all designed compounds are also available as a Source Data File and upon reasonable request by the corresponding authors.
Data exclusions	Extreme outliers in in vitro assays were identified and cautiously excluded, otherwise, data was kept as complete as possible.
Replication	Data were replicated using at least three biologically independent replicates. Binding assays were performed in duplicates and functional assays in triplicates. See figure and table legends for specific details.
Randomization	Ligands tested in vitro were selected based on their ability to pass interface metrics, thus no randomization was performed. However, covariates were controlled through careful matching of experimental groups based on relevant variables. This approach allowed us to reduce the potential confounding effects of covariates and improve the internal validity of our findings. For functional and binding assays, randomization is not relevant as no group allocations were performed. For the behavioral studies, mice were randomly assigned across treatments.
Blinding	The in vitro characterization of designed ligands was performed unblinded. Blinding was not implemented in the in vitro pharmacology study due to the highly quantitative and automated nature of the experiments, where subjectivity and bias were minimal. The large number of samples and controlled conditions also made the logistics of blinding impractical. The study aimed for high reproducibility and validation of assay protocols, and the experimental design itself was structured to minimize potential sources of bias. However, the affinity, potency and efficacy as well as receptor selectivity data were confirmed at three independent labs. The in vivo experiments in mice were performed in a blinded manner.

Reporting for specific materials, systems and methods

We require information from authors about some types of materials, experimental systems and methods used in many studies. Here, indicate whether each material, system or method listed is relevant to your study. If you are not sure if a list item applies to your research, read the appropriate section before selecting a response.

Materials & experimental systems

n/a	Involved in the study	
[ ]	[x]	Antibodies
[ ]	[x]	Eukaryotic cell lines
[x]	[ ]	Palaeontology and archaeology
[ ]	[x]	Animals and other organisms
[x]	[ ]	Clinical data
[x]	[ ]	Dual use research of concern
[x]	[ ]	Plants

Methods

n/a	Involved in the study	
[x]	[ ]	ChIP-seq
[x]	[ ]	Flow cytometry
[x]	[ ]	MRI-based neuroimaging

Antibodies

Antibodies used	gp64-PE antibody (expression system, #97-201), anti-FLAG-horseradish peroxidase-conjugated antibody (Sigma, A8592)
Validation	gp64-PE antibody was purchased from Expression Systems and was used for baculovirus titration. The detailed information can be found at https://expressionsystems.com/product/gp64-pe-antibody/<br>anti-FLAG-horseradish peroxidase-conjugated antibody was ordered from Sigma-Aldrich and was used in ELISA experiment for the detection of KOR receptor (wt and mutants) expression level. The detailed information can be found at https://www.sigmaaldrich.com/US/en/product/sigma/a8592

Eukaryotic cell lines

Policy information about cell lines and Sex and Gender in Research

Cell line source(s)	Human embryonic kidney cells 293 (HEK293T) and chinese hamster ovarian cells (CHO-K1) were obtained from American Type Culture Collection (ATCC). Spodoptera frugiperda (Sf9) cells are from Expression Systems (#94-001S).
Authentication	All cells used in this study are commercial and were obtained from vendors as indicated in the manuscript. HEK293T and CHO-K1 were certified by ATCC using morphology and growth characteristics as well as STR profiling. Sf9 cells are commercial and obtained from vendors as indicated in the manuscript. No additional authentication was performed by the authors of this study.
Mycoplasma contamination	Mycoplasma contamination was tested prior to conducting in vitro assays and ruled out for cell lines used in the study.
Commonly misidentified lines<br>(See ICLAC register)	No commonly misidentified cell lines were used in the study.

Animals and other research organisms

Policy information about studies involving animals; ARRIVE guidelines recommended for reporting animal research, and Sex and Gender in Research

Laboratory animals	Experiments were performed in male SWISS mice (RjOrl:SWISS; 8-10 weeks old, 30-35 g body weight) purchased from Janvier Labs (Le Genest-Saint-Isle, France). Mice were group-housed in a temperature- (21-22°C) and humidity-controlled (60-70%) specific pathogen free room with a 12 h light/dark cycle and with free access to food and water. All behavioral experiments were performed during the light cycle.
Wild animals	No wild animals were used in these study.
Reporting on sex	We used male mice to establish pain and behavioral responses as these types of studies are commonly reported using either male or female mice, or both sexes.
Field-collected samples	This study did not invole samples collected from the field.
Ethics oversight	All animal care and experimental procedures were in accordance with the ethical guidelines for the animal welfare standards of the European Communities Council Directive (2010/63/EU) and were approved by the Committee of Animal Care of the Austrian Federal Ministry of Science and Research (Protocol Nr. 2022-0.159.599).

Note that full information on the approval of the study protocol must also be provided in the manuscript.

Plants

Seed stocks	n.a.
Novel plant genotypes	n.a.
Authentication	n.a.
